# Symbiosis of Carpenter Bees with Uncharacterized Lactic Acid Bacteria Showing NAD Auxotrophy

**DOI:** 10.1128/spectrum.00782-23

**Published:** 2023-06-22

**Authors:** Shinji Kawasaki, Kaori Ozawa, Tatsunori Mori, Arisa Yamamoto, Midoriko Ito, Moriya Ohkuma, Mitsuo Sakamoto, Minenosuke Matsutani

**Affiliations:** a Department of Molecular Microbiology, Tokyo University of Agriculture, Tokyo, Japan; b Microbe Division/Japan Collection of Microorganisms, RIKEN BioResource Research Center, Tsukuba, Japan; c NODAI Genome Research Center, Research Institute, Tokyo University of Agriculture, Tokyo, Japan; University of Valencia

**Keywords:** carpenter bees, honey bees, gut microbiome, lactic acid bacteria, bifidobacteria, NAD biosynthesis

## Abstract

Eusocial bees (such as honey bees and bumble bees) harbor core gut microbiomes that are transmitted through social interaction between nestmates. Carpenter bees are not eusocial; however, recent microbiome analyses found that *Xylocopa* species harbor distinctive core gut microbiomes. In this study, we analyzed the gut microbiomes of three *Xylocopa* species in Japan between 2016 and 2021 by V1 to V2 region-based 16S rDNA amplicon sequencing, and 14 candidate novel species were detected based on the full-length 16S rRNA gene sequences. All *Xylocopa* species harbor core gut microbiomes consisting of primarily lactic acid bacteria (LAB) that were phylogenetically distant from known species. Although they were difficult to cultivate, two LAB species from two different *Xylocopa* species were isolated by supplementing bacterial culture supernatants. Both genomes exhibited an average LAB genome size with a large set of genes for carbohydrate utilization but lacked genes to synthesize an essential coenzyme NAD, which is unique among known insect symbionts. Our findings of phylogenetically distinct core LAB of NAD auxotrophy reflected the evolution of *Xylocopa*-restricted bacteria retention and maintenance through vertical transmission of microbes during solitary life. We propose five candidate novel species belonging to the families Lactobacillaceae and Bifidobacteriaceae, including a novel genus, and their potential functions in carbohydrate utilization.

**IMPORTANCE** Recent investigations found unique microbiomes in carpenter bees, but the description of individual microbes, including isolation and genomics, remains largely unknown. Here, we found that the Japanese *Xylocopa* species also harbor core gut microbiomes. Although most of them were difficult to isolate a pure colony, we successfully isolated several strains. We performed whole-genome sequencing of the isolated candidate novel species and found that the two Lactobacillaceae strains belonging to the *Xylocopa*-specific novel LAB clade lack the genes for synthesizing NAD, a coenzyme central to metabolism in all living organisms. Here, we propose a novel genus for the two LAB species based on very low 16S rRNA gene sequence similarities and genotypic characters.

## INTRODUCTION

The gut microbial communities of insects have wide variation and are known to be highly specialized based on host species. Many studies have been performed that identified host-specific microbiota of insects and showed obligate mutualism between the host and bacterial species (1–4). Insect symbionts are mainly classified as obligatory and facultative. Obligate symbionts have become highly dependent on insect hosts and either have highly reduced genome sizes or carry abundant pseudogenes compare to their free-living relatives (4–6). Although gut microbes of corbiculate bees are not obligate symbionts, these host-adapted symbionts have been found to be involved in digestion, metabolism, the immune system, and pathogen resistance. However, the role of microbiomes and the mechanism of symbiosis associated with host specificity remains to be clarified.

Gut microbiomes of the family Apidae have been extensively examined in eusocial bees such as honey bees and bumble bees, and are composed of bee-specific core taxa, including members of the genus *Lactobacillus*, such as Firm-4, Firm-5, *Bifidobacterium*, and *Bombiscardovia*, and some Gram-negatives, such as *Apibacter*, *Gilliamella*, and *Snodgrassella* species (7–10). Although these core taxa were shown to potentially vary and have unique abundance patterns depending on individual host species and sometimes geography, these bacterial species are extremely specialized to the corbiculate bee gut and have not been found elsewhere (11, 12).

In contrast to eusocial bees, the microbiomes of solitary bees are thought to be highly variable and heterogenous because the microbes are predominantly sourced from the environment (13–16). Carpenter bees are distributed worldwide; approximately 500 species are known, and they are the effective pollinators of diverse crops (17, 18). *Xylocopa* species are traditionally considered solitary bees but show facultatively or incipiently social behavior, where a female bee feeds younger nestmates via trophallaxis (18–22). They usually make nests by tunneling into old timber and bamboo with a small social group (17, 20). Most species occur in the tropics or subtropics, and limited *Xylocopa* species are known to be distributed in Japan (23–27). Recent investigations of microbiomes in *Xylocopa* species in North America suggested the existence of a microbiome compositions that were distinct from those of other eusocial and solitary bees (28–30). To the best of our knowledge, most of these bacteria have not been isolated, and two novel species, *Bifidobacterium xylocopae* and *Bifidobacterium aemilianum*, were isolated as *Xylocopa*-specific bifidobacteria in Italy (31).

During our investigation of microbiome interactions between flowers and flower-visiting insects, the flower specific anaerobic lactobacilli *Holzapfeliella floricola* (originally named Lactobacillus floricola) and Apilactobacillus ozensis (originally named Lactobacillus ozensis), were isolated and characterized (32–34). We also investigated the gut microbiomes of flower-visiting bees living in Japan. The V1 to V2 region-based 16S rRNA gene amplicon sequencing revealed *Xylocopa*-specific core lactic acid bacteria (LAB) across species, years, and seasons, and several strains were isolated and identified as novel species. Genome analysis revealed that the core strains lacked NAD biosynthesis; however, the genome size and number of coding sequences were not significantly reduced, which is a rare character among not only known LAB but also obligate symbionts in insects (4, 6, 35). In addition, through taxonomic investigations, we proposed five candidate novel species belonging to the families Lactobacillaceae and Bifidobacteriaceae.

## RESULTS AND DISCUSSION

### *Xylocopa*-specific microbiota identification.

**FIG 1:**
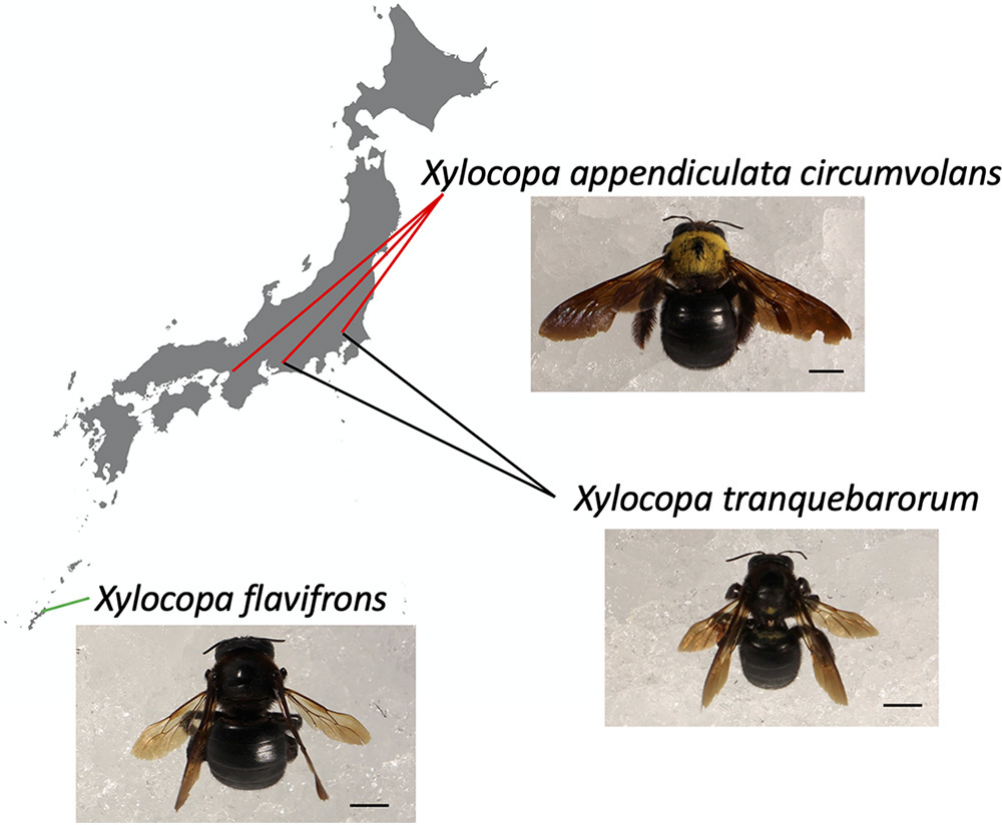
Distribution and sample collection sites of *Xylocopa* species in Japan. *Xylocopa appendiculata circumvolans* and *X. tranquebarorum* are distributed on the main island of Japan, and *X. flavifrons* is distributed on the main island of Okinawa Prefecture. *X. a. circumvolans* and *X. flavifrons* nest in wood, and *X. tranquebarorum* nests in bamboo. Scale bar, 0.5 cm.

During our investigation of the gut microbiota of flower-visiting insects between 2016 and 2021 in Japan, we collected 29 *X. appendiculata circumvolans* samples, two *X. flavifrons* samples, nine *X. tranquebarorum* samples, 16 Apis mellifera samples, and 11 bumble bee (Bombus terrestris, *Bombus hypocrite*, *and Bombus ardens ardens*) samples (Fig. 1; Fig. S1; Table S1), and performed microbiome analysis by V1 to V2 region-based 16S rRNA gene amplicon sequencing, which is optimal for species-level analysis. Gut microbial communities were profiled at a depth of 43,840 reads per sample. We obtained 4,227 qualified amplicon sequence variants (ASVs), and the carpenter bee gut microbiome was analyzed by QIIME2.

The obtained gut microbiomes of *Xylocopa* samples were mainly composed of LAB with low sequence similarity to known LAB species. Therefore, we attempted to isolate *Xylocopa*-specific LAB from these samples using cultivation methods. Bee samples were cooled on ice and the entire guts were removed and subjected to cultivation using MRS medium. Anaerobically grown colonies were randomly selected and investigated using a partial 16S rRNA gene sequence. The full-length 16S rRNA sequences were obtained by PCR amplification for 10 candidate novel species of Lactobacillaceae (Kim32-2, KimG, KimC2, KimE, KimD, XA1, XA2, XA3, XA4, XA14), three of Bifidobacteriaceae (Kim37-2, KimA, KimH), and one of *Entomomonas* (XA13). These individual sequences were used for further taxonomic analysis.

**FIG 2:**
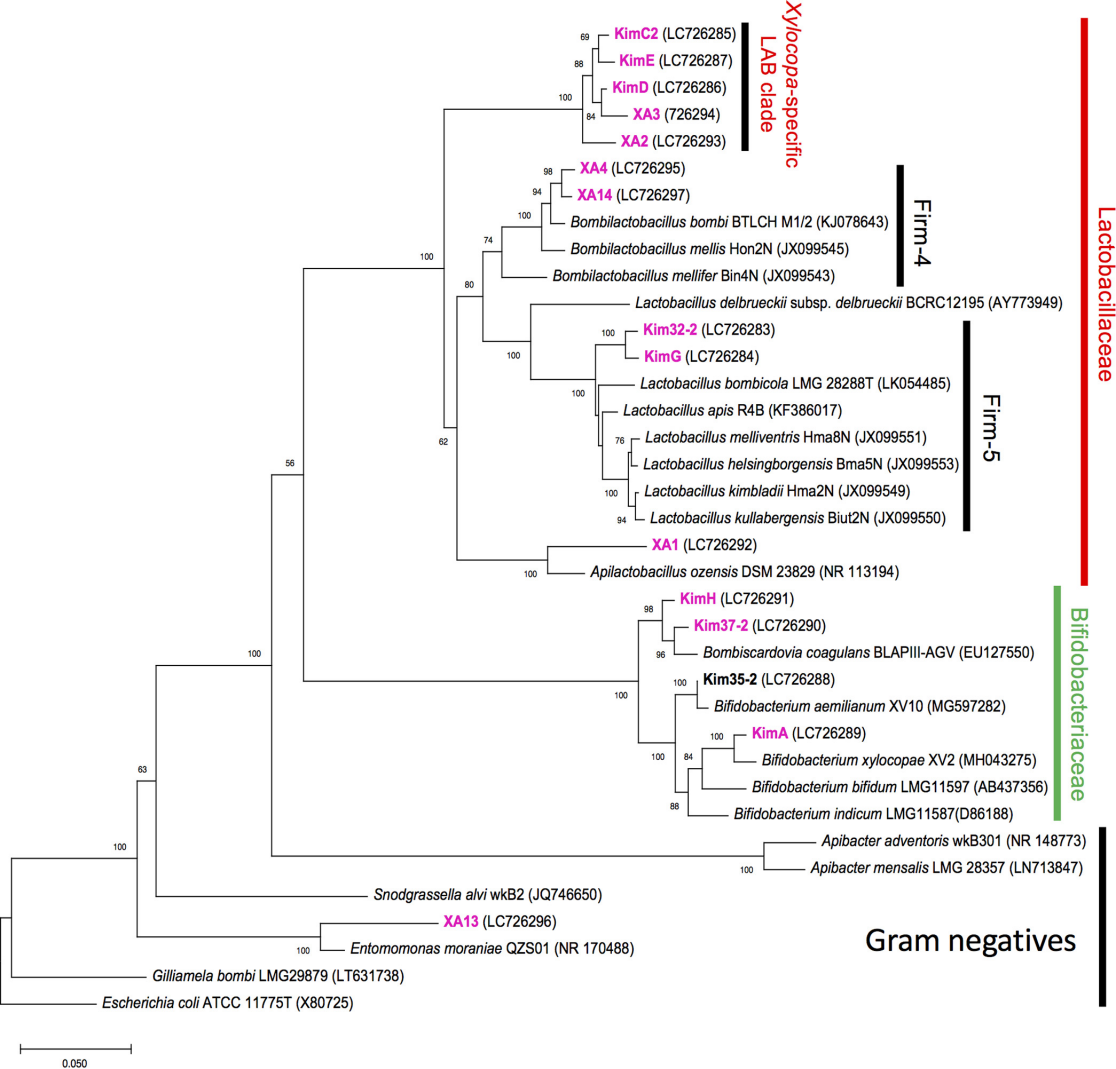
Phylogenetic tree showing the gut microbiota of *Xylocopa* species. The tree was constructed using the maximum likelihood method based on the full-length 16S rRNA gene sequences. Bootstrap values above 50% are shown at branching points. Candidates of novel species of isolates are shown in pink font. Accession numbers are shown in parenthesis.

**FIG 3:**
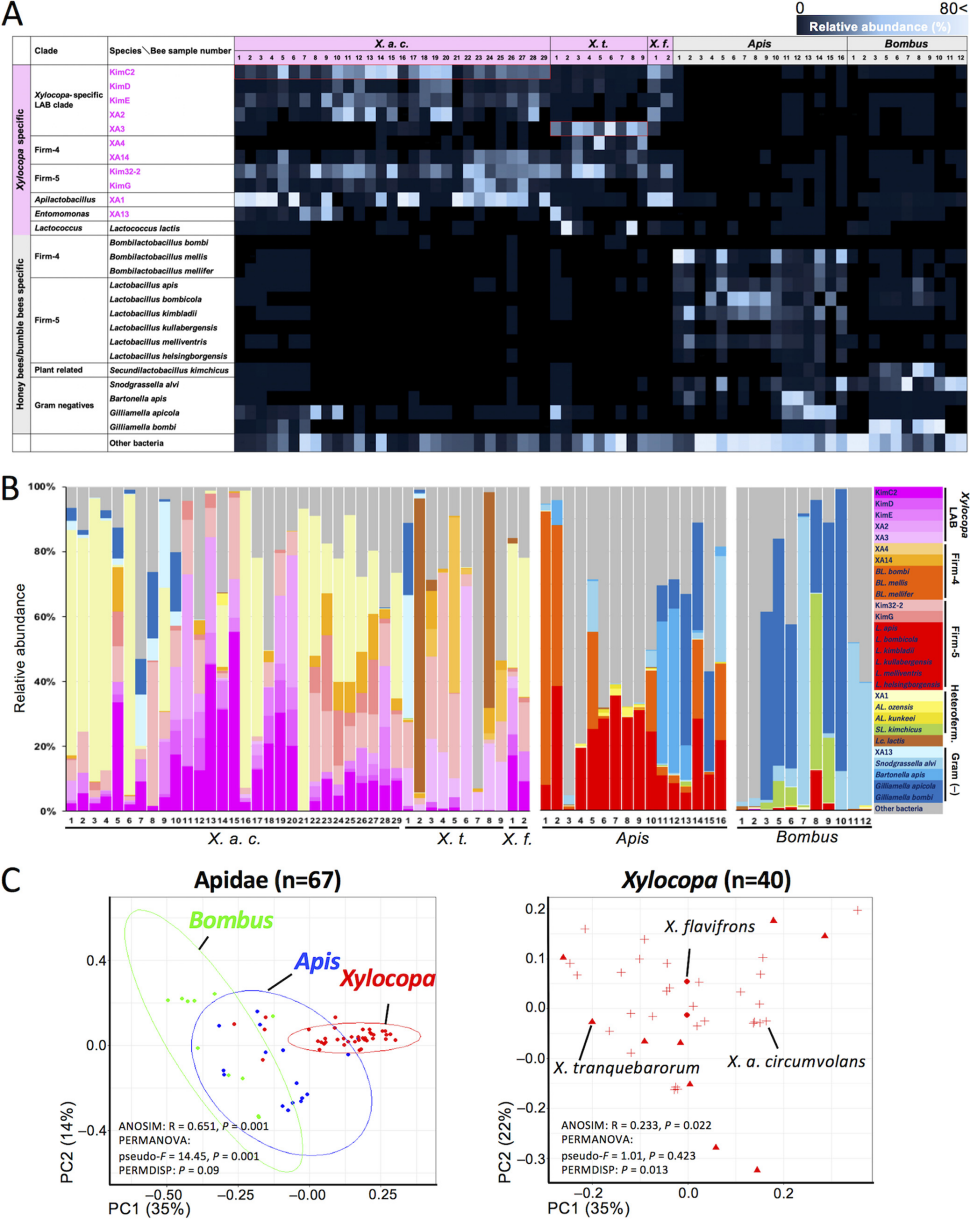
Comparison of the gut microbial communities of *Xylocopa* and eusocial bees. (A) Gut microbial community profiles of *Xylocopa* species compared with those of eusocial bees (honey bees and bumble bees), some of which were collected at the same sampling area. ASVs that showed BLASTN >97% identity to the 16S rRNA gene sequence of known species, including 14 candidate novel species were assigned taxon names and mapped for each species as relative abundance for each bee sample. The candidate novel species detected in this study are shown in pink font. *X. a. c.*: *Xylocopa appendiculata circumvolans*, *X. t.*: *X. tranquebarorum*, *X. f.*: *X. flavifrons.* (B) Relative abundance of ASVs, including taxon names assigned in panel A. Candidate novel species that belong to *Xylocopa*-specific novel LAB clade were shown in pink gradient color bars. *Bombilactobacillus* (*BL*.); *Apilactobacillus* (*AL.*); *Secundilactobacillus* (*SL.*); *Lactococcus* (Lc.); heterofermentative LAB clade (Heteroferment.); Gram-negative species {Gram (-)}. (C) Principal coordinates analysis (PCoA) of the gut microbial communities. Left, PCoA using all samples showing clear separation between the three different Apidae families. Right, PCoA using all *Xylocopa* samples showing unclear separation between the three different *Xylocopa* species. n, number of bee samples used for analysis. Circles indicate the 95% confidence intervals.

Fig. 2 shows phylogenies based on the full-length 16S rRNA gene sequences (Fig. S2 and S3). Fig. 3 shows gut microbial community comparison between *Xylocopa* species and eusocial bees based on ASVs. ASVs corresponding to taxon Kim32-2 were detected in all surveyed *Xylocopa* species (Fig. 3A and B). Phylogenetic analysis placed Kim32-2 in a sister clade of Firm-5 species; therefore, we include this clade Firm-5 (Fig. 2). ASVs corresponding to taxon KimC2 were abundant in *X. a. circumvolans* and *X. flavifrons* (Fig. 3A and B). Similarly, ASVs corresponding to taxon XA3 were primarily detected in *X. tranquebarorum* and were rarely present in *X. a. circumvolans* (Fig. 3A and B). KimC2 and XA3 were close relatives based on phylogenetic analysis (described later); therefore, the differences in their distributions may reflect host specificity. Taxon KimC2 and XA3 comprised a *Xylocopa*-specific clade, and five different taxa were detected in this clade (Fig. 2). These *Xylocopa*-specific ASVs were rarely detected in the bumble bees and honey bees (Fig. 3A and B). The full-length 16S rRNA gene sequences of five taxa showed less than 90% identity to known species; therefore, we named this clade as a *Xylocopa*-specific LAB clade (Fig. 2). Taxon XA1 was also detected as one of the most abundant ASVs in *X. a. circumvolans* and *X. flavifrons* but rare in *X. tranquebarorum*, honey bees, and bumble bees. XA1 belongs to the clade of heterofermentative LAB group, which includes *Apilactobacillus kunkeei* and *A. ozensis* isolated from fructose-rich sources such as flowers and bee hives (33, 35).

ASVs corresponding to *Snodgrassella*, *Gilliamella*, *Lactobacillus* Firm-4 (Bombilactobacillus mellis, Bombilactobacillus mellifer, and *Bombilactobacillus bombi*), and *Lactobacillus* Firm-5 (*L. apis*, *L. bombicola*, *L. kimbladii*, *L. kullabergensis*, *L. melliventris*, and *L. helsingborgensis*) are prevalent, especially in honey bees, and they have been extensively characterized in honey bees and bumble bees (9, 10, 12, 13, 36–40). These core microbes were also detected in our samples of honey bees, and these core species were rarely found (except *Gilliamella*) in *Xylocopa* species. The absence of *Snodgrassella* was also reported in *Xylocopa* species inhabiting North America (29, 30). Principal coordinates analysis (PCoA) showed clear separation of gut microbiota composition in *Xylocopa* species compared with honeybees and bumblebees, but clear separation was not detected among the microbiota of *Xylocopa* species (Fig. 3C). Overall, although the gut microbiomes of *Xylocopa* species and other tested eusocial bees showed phylogenetic relationships, the distributions of ASVs were separated between *Xylocopa* and other bees and between *X. a. circumvolans* and *X. tranquebarorum* (Fig. 3A and B).

### LAB isolation and characterization.

Some colonies obtained on the first culture plate were difficult to further cultivate in the subculture plates but successfully developed by supplementing with other bacterial culture-supernatants. The obtained pure isolates showed very low 16S rRNA gene sequence identity to known species, and they were separated into two bacterial lineages (KimC2 and XA3 lineages) based on the 16S rRNA gene sequences. Two strains, KimC2 and XA3 isolated from *X. a. circumvolans* and *X. tranquebarorum*, respectively, were chosen as the type strain for further characterization.

Phylogenetic analysis placed the two type strains (KimC2 and XA3) in a *Xylocopa*-core clade, and each showed 97.64% identity of the full-length 16S rRNA gene sequence. The growth of KimC2 and XA3 demonstrated that they were strictly anaerobic. 16S rRNA gene sequences of KimC2 and XA3 showed 88.18% and 88.03% identity to *Lacticaseibacillus saniviri* JCM 17471^T^, respectively, and approximately 85% to 88% identity to phylogenetically related species such as *Bombilactobacillus bombi* DSM 26517^T^, Pediococcus pentosaceus DSM 20336^T^, and *Lacticaseibacillus pantheris* LMG 21017^T^. Kim32-2 developed a single colony under anaerobic conditions and its 16S rRNA gene sequence was phylogenetically placed in a *Lactobacillus* Firm-5 clade (Kim32-2 showed 95.2% identity to *L. bombicola* H70-3). The associated bifidobacteria on MRS plates were also isolated and named Kim37-2, Kim35-2, and KimH. The 16S rRNA gene sequences of the strains Kim37-2 and KimH showed 96.78% and 96.88% identity to Bombiscardovia coagulans DSM 22924^T^, respectively. Kim35-2 showed 99.5% identity to *Bifidobacterium aemilianum* DSM 104956^T^ isolated from Italian *Xylocopa* (31). Kim37-2 and KimH could develop colonies on agar plates under air conditions similar to the genus *Bombiscardovia* (Fig. S5B). Therefore, these species should be separated from the genus *Bifidobacterium*.

### Complete genome analysis of the isolates.

**FIG 4:**
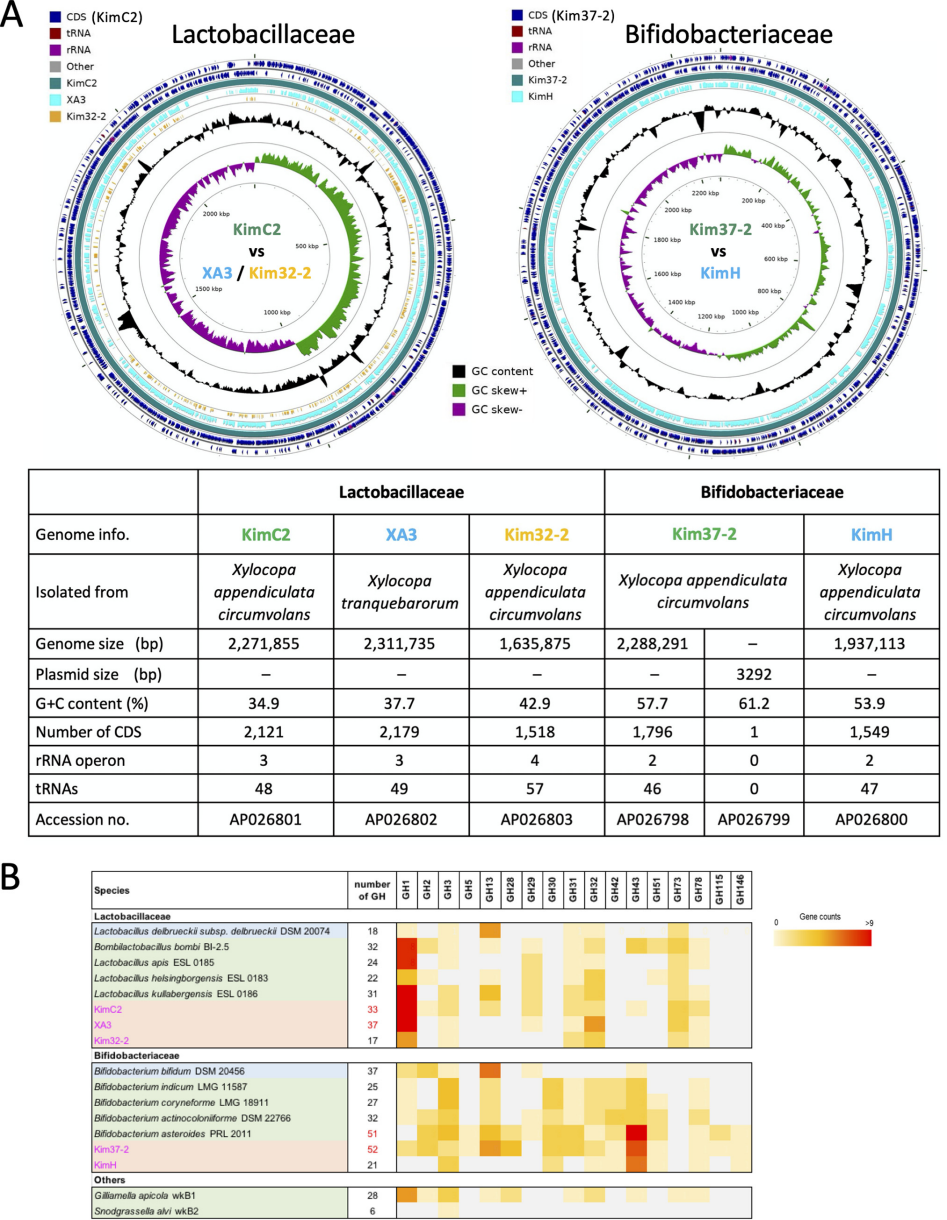
Graphical circular maps of novel isolates that belong to Lactobacillaceae and Bifidobacteriaceae. (A) Genome sequences of Lactobacillaceae KimC2, XA3, and Kim32-2, and of Bifidobacteriaceae Kim37-2 and KimH were mapped separately using PROmer and the circular maps were generated using CGView. The outer blue rings displayed the genes and features of KimC2 (Lactobacillaceae) or Kim37-2 (Bifidobacteriaceae) on both strands. Light blue and brown rings illustrate the BLAST results when the genome sequences of XA3 and Kim32-2 (Lactobacillaceae) or KimH (Bifidobacteriaceae) were compared with those of KimC2 or Kim37-2, respectively. The inner two rings display GC content (black) and GC skew (green and purple) for each chromosome. (B) Heatmap showing the distribution of the identified glycoside hydrolase (GH) families across the analyzed genomes. Strains are colored for type species of the genus (blue), honey bee strains (green), and *Xylocopa* strains (pink).

Whole-genome sequencing of five candidate novel species (KimC2, XA3, Kim32-2, KimH, and Kim37-2) was performed using PacBio Sequel or RSII sequencing analyses. *De novo* assembly was performed using the hierarchical genome assembly process (Table S11). The complete genome sizes with sequence information of each isolate are shown in Fig. 4A. Each single contig representing one chromosome, and a plasmid was detected in Kim37-2. The genome sizes of KimC2 and XA3 (2.27 Mbp and 2.31 Mbp) were determined to be approximately the average genome size of lactobacilli compared with the smallest (*Fructilactobacillus sanfranciscensis*, 1.23 Mbp), largest (*Lentilactobacillus parakefiri*, 4.91 Mbp), Firm-4 (*Bombilactobacillus*, 1.81 to 1.84 Mbp), Firm-5 (*L. apis*, *L. bombicola*, *L. helsingborgensis*, *L. kimbladii*, *L. melliventris*, and *L. kullabergensis*,1.64 to 2.19 Mbp), and type species (Lactobacillus delbrueckii subsp. *delbrueckii*, 1.76 Mbp) (35). The digital DNA–DNA hybridization (dDDH), average nucleotide identity (ANI), and average amino acid identity (AAI) of KimC2, XA3, Kim32-2, Kim37-2, and KimH to known species are summarized in Table S2 to S7. The five candidates of novel species showed less than 75% ANI values with other related taxa. In addition, the dDDH values and the AAI values between the five strains and other related taxa ranged from 18.2% to 31.0% and 48.2% to 77.6%, respectively. These values are lower than the species boundary (35, 41, 42). The ANI, dDDH, and AAI values between KimC2 and XA3 (75.0%, 20.0%, and 73.8%, respectively), and between KimH and Kim37-2 (76.4%, 22.1%, 77.6%, respectively) were also lower than the species boundary. Therefore, we proposed that these strains are novel species.

**FIG 5:**
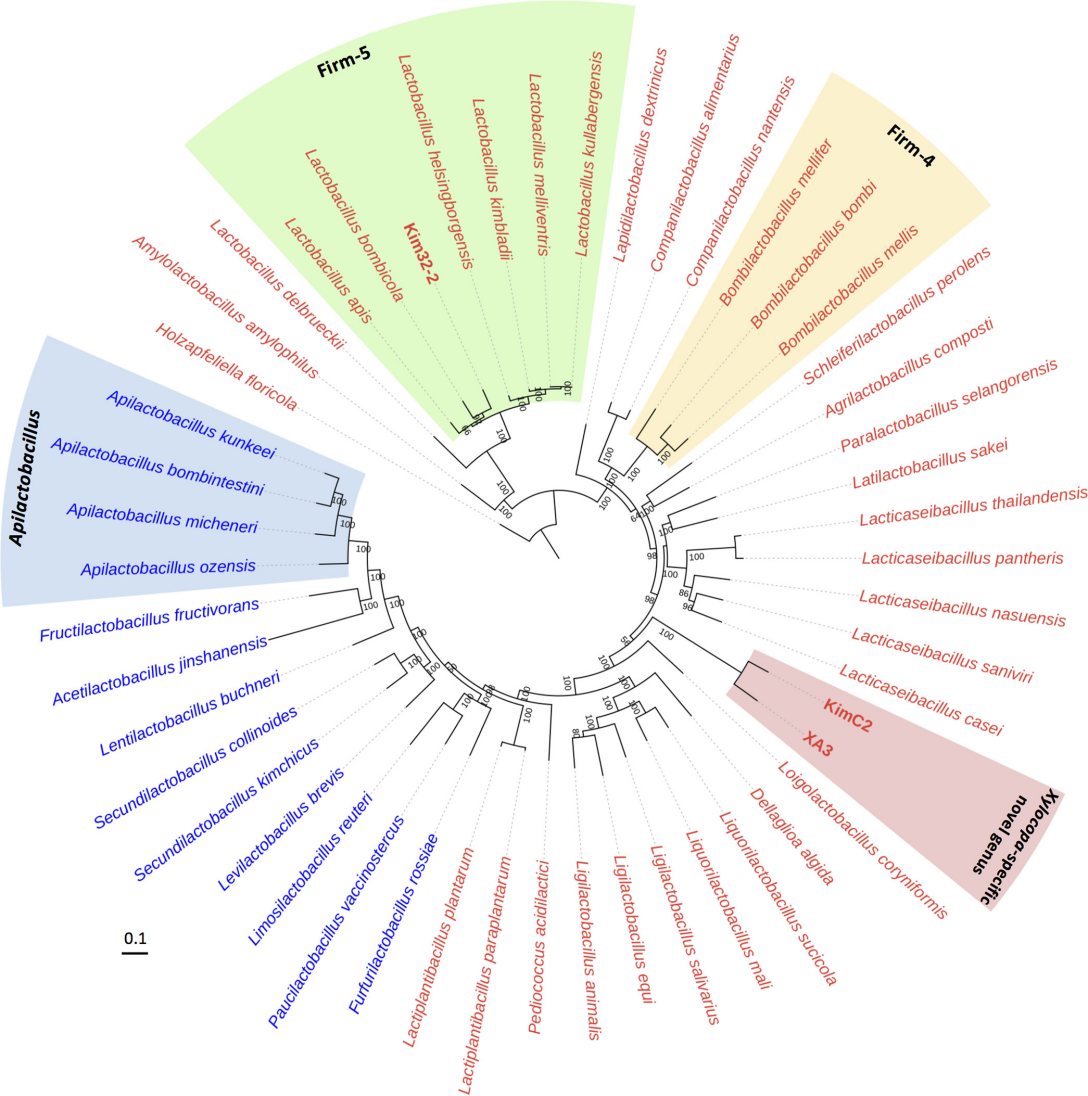
Core genome phylogenetic tree of Lactobacillaceae species. Phylogenetic tree consisting of 50 strains, including the 26 type strains of the 26 phylogenetic groups of the Lactobacillaceae (35) with honey bee specific Lactobacillaceae species (from *Apilactobacillus*, Firm-4, and Firm-5) and with candidates of three novel species (KimC2, XA3, and Kim32-2) including their closest relatives based on 16S rRNA gene similarity. The phylogenetic analysis was based on the concatenated alignment of protein sequences for the 114 single-copy core genes. The maximum likelihood tree was inferred by RAxML using the best model (LG + I + G + F). Bootstrap support values were calculated from 500 replicates, and values above 50% are labeled. Names of homofermentative species are shown in red; names of heterofermentative species are shown in blue. The candidate novel species are shown in Bold. *Holzapfeliella floricola* JCM 16512^T^ was used as an outgroup.

To determine the phylogenetic positions of the strain KimC2, XA3, and Kim32-2, a core genome phylogenetic tree was constructed using 50 strains, including 26 type strains representing 26 phylogenetic groups of Lactobacillaceae (35), phylogenetic relatives of the isolates, and the bee-specific species, including *Lactobacillus* Firm-4 and Firm-5 from honey bees and bumble bees (Fig. 5). The phylogenetic positions of 26 type strains were consistent with those of the previous study, and KimC2 and XA3 formed a monophyletic clade in Lactobacillaceae. Kim32-2 was placed in the *Lactobacillus* Firm-5 clade.

**FIG 6:**
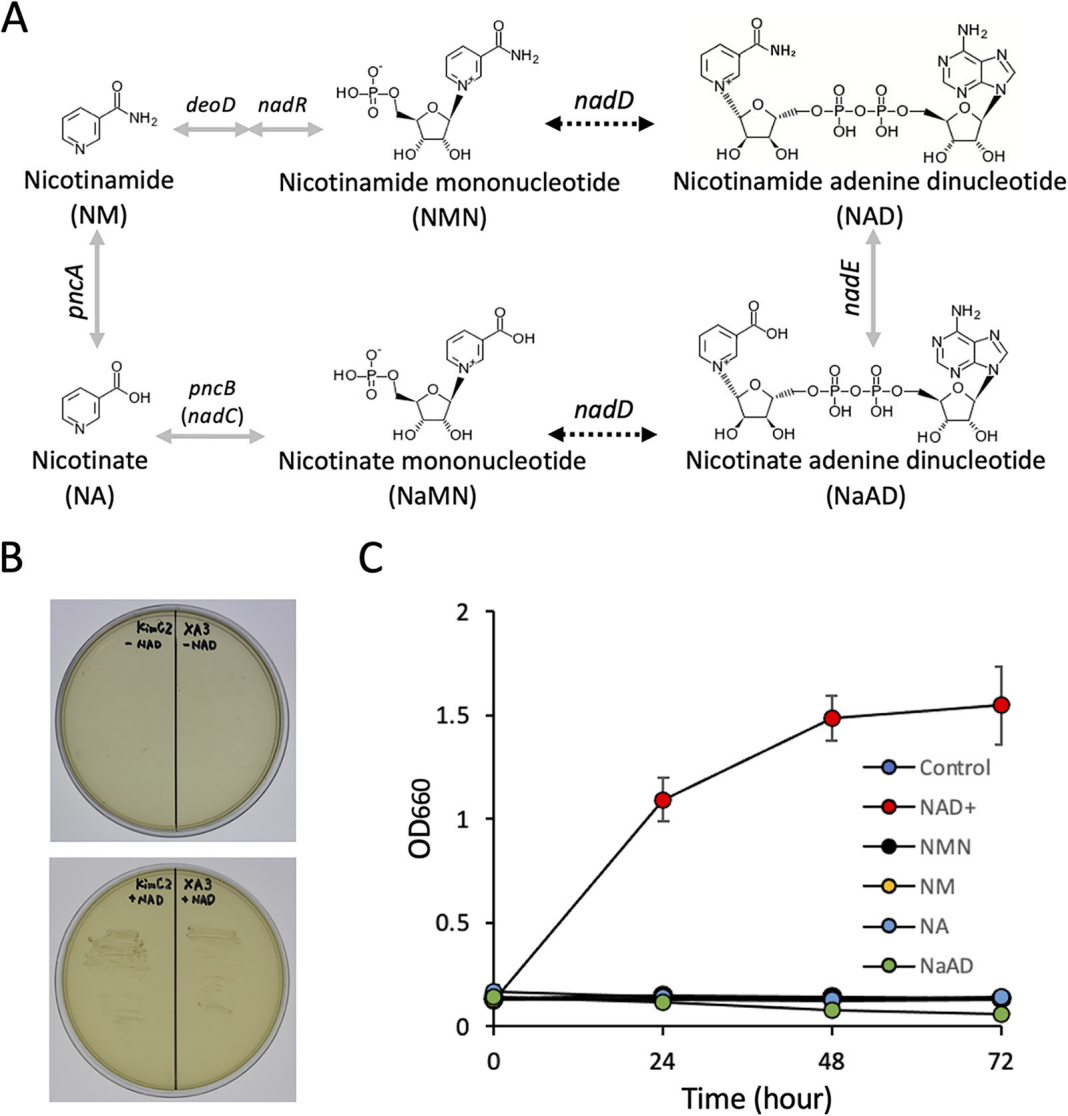
KimC2 and XA3 lack the genes for NAD biosynthesis. (A) Genome sequence of KimC2 and XA3 revealed a lack of NAD biosynthesis genes, which are indicated by gray arrows. Dot arrows indicate the existence of potential genes encoding homologs. (B) Growth of KimC2 and XA3 with and without NAD^+^ supplement in MRS plates. (C) Growth of KimC2 in the absence or presence of nicotinate compounds in MRS medium. Data represent the average ± SD of three independent experiments.

Using the auto-annotation tools of Clusters of Orthologous Groups (COG) and DFAST in combination with the Kyoto Encyclopedia of Genes and Genomes (KEGG) mapper, KimC2 and XA3 were shown to lack the genes for NAD biosynthesis (Fig. 6A), specifically the genes for nicotinate phosphoribosyltransferase (*pncB* or *nadC*) and NAD^+^ synthetase (*nadE*) (two genes comprise an operon in some LAB species, and both genes are well conserved in Lactobacillaceae species: e.g., *L. apis* WP_109715170 and WP_109715171, respectively). To the best of our knowledge, strains that show NAD auxotrophy have not been isolated in Lactobacillaceae species (35).

### Phenotypic and chemotaxonomic studies on the isolated strains.

We performed chemotaxonomic studies on the proposed novel species to infer their role in carbohydrate utilization, their fermentation profile, and their morphology.

KimC2 and XA3 were isolated from single colonies developed on MRS plates supplemented with supernatants of culture medium from other bacteria. Genome analysis revealed that KimC2 and XA3 lacked genes encoding enzymes for NAD biosynthesis (43–45). Although genome analyses of KimC2 and XA3 revealed the presence of *nadD* gene ortholog (KimC2_07880 and XA3_07560, respectively) of which the gene product (nicotinate-nucleotide adenylyltransferase) catalyzes the reversible adenylation of nicotinate mononucleotide (NaMN) to nicotinate adenine dinucleotide (NaAD) even though the similarity to the orthologs in phylogenetically related species were low (e.g., KimC2_07880 showed 34% identity to NadD of *Bombilactobacillus bombi* WP_118901107 and 27.9% identity to functionally characterized NadD of Streptococcus pneumoniae SP_1747) (44). Further, although KEGG mapper showed the lack of *pncA*, *deoD*, and *nadR* genes in the genomes of KimC2 and XA3, the functions of these genes are not well characterized in Lactobacillaceae, and there might be some unknown salvage pathways for synthesizing NAD cofactor in microbes (46). Therefore, we tested the auxotrophy in the presence of nicotinamide-related compounds.

The growth of both strains recovered only by supplementation of NAD(H) and NADP(H) but not nicotinamide (NM), nicotinate (NA), nicotinamide mononucleotide (NMN), and nicotinate adenine dinucleotide (NaAD) in MRS medium (Fig. 6B and C). These results confirmed the specific deletion of NAD biosynthesis pathway in KimC2 and XA3. Cultivation of *Xylocopa* gut samples on MRS agar plates supplemented with NAD produced single colonies carrying 16S rRNA gene sequences of KimC2 and XA3 lineages; however, we did not successfully obtain pure single colonies that belonged to KimE, KimD, and XA2 in a *Xylocopa*-core clade (Fig. 2). We are currently attempting to obtain pure single colonies of these species using other cultivation methods by estimating the possibility that other mutations exist.

Fermentation products, carbohydrate utilization, and fatty acid methyl ester composition are listed in Table S8 to S10. Lactobacillaceae KimC2 and XA3 showed the ability to utilize various carbohydrates, which was also suggested from the genome analysis in which the genes for carbohydrate utilization and PTS transporter were shown to be abundant (Fig. S4). Using the Carbohydrate-Active Enzyme (CAZy) database (47), we investigated the presence of genes for carbohydrate utilization (Fig. 4B; Data set S1). The number of glycoside hydrolase (GH) gene families included orthologs of potential glucosidases, galactosidases, mannosidases, fucosidases, rhamnosidases, xylosidases (e.g., GH1-3, GH31, GH43, GH78), and invertase (GH32) in Lactobacillaceae KimC2 and XA3, which were similar to those of *Lactobacillus* Firm-5 (10). Therefore, we infer that one of the functions of KimC2 and XA3 is to uptake a variety of sugars for fermentation like Firm-5. Bifidobacteriaceae Kim37-2 and KimH were also rich in genes in the category of carbohydrate utilization and transport; however, both strains showed poor ability to utilize monosaccharides (Table S9). Genome analysis revealed that both strains carried commonly the genes encoding orthologs for potential polysaccharide digestion, such as GH13 (amylase), GH28 (rhamnogalacturonase), and GH43 (arabinofuranosidase), which were similar to those in the honey bee strain *B. asteroides* (9) (Fig. 4B). With regard to oxidative growth, Kim37-2 and KimH, which belonged to Bifidobacteriaceae, were microaerophilic (Fig. S5B); however, KimC2, XA3, and Kim32-2, which belonged to Lactobacillaceae, were strictly anaerobic. Kim37-2 and KimH carry anti-oxidative enzymes such as superoxide dismutase (found in Kim37-2 and KimH) and catalase (found in Kim37-2) like honey bee bifidobacteria (48, 49), while these genes are not found in the genomes of Lactobacillaceae isolates. These results suggested that the core-LAB strains are involved in lactic acid fermentation using various carbohydrates under strict anaerobic conditions. The relative abundances of ASVs corresponding to taxa KimC2 and XA3 vary from 1% to 70% even in the *Xylocopa* samples collected in the same sampling area; however, the total abundances of ASVs corresponding to LAB, including *Xylocopa* core strains, XA1, XA4, and XA14, account for more than 50% in most *Xylocopa* samples. These results suggest that the variation in each LAB population may be influenced by several factors such as foods, metabolites, and O_2_ tension in the gut.

To summarize, we isolated two novel LAB species that showed auxotrophy for NAD. Interestingly, although obligate symbionts usually shared reduced genomes that lack genes in almost all functional categories, the genome size of the NAD auxotrophs KimC2 and XA3 showed average genome sizes of free-living LAB, and all categories of genes were normal or rather high in carbohydrate utilization (Fig. S4). These results indicated that the isolated two strains can reproduce independently when NAD is provided under symbiosis conditions.

Although the merit of acquisition of the NAD auxotroph LAB in *Xylocopa* is largely unknown, a phylogenetic distance of these core LAB that cannot reproduce themselves suggested that the symbiont acquisition is ancient and specific, and this may be maintained by maternal transmission in the unique lifestyle of carpenter bees. Still, several strains have not yet been isolated to identify specific functions in *Xylocopa*. Further isolation and characterization will help clarify carpenter bee ecology, reproduction, and evolution.

### Taxonomy.

**(i) Description of *Xylocopilactobacillus* gen. nov.** *Xylocopilactobacillus* (Xy.lo.co.pi.lac.to.ba.cil’lus. N.L. fem. n. *Xylocopa*, a genus of carpenter bees; N.L. masc. n. *Lactobacillus*, a bacterial genus; N.L. masc. n. *Xylocopilactobacillus*, a *Lactobacillus* from *Xylocopa* bees). *Xylocopilactobacillus* species have been isolated from the gut of carpenter bees in Japan. Gram-positive, rod-shaped, catalase negative, homofermentative, and G+C content ranging from 34.9 to 37.7. The type species of the genus is *Xylocopilactobacillus apis*.

**(ii) Description of *Xylocopilactobacillus apis* sp. nov.** *Xylocopilactobacillus apis* (a’pis. L. gen. n. *apis*, of a bee). Cells are Gram-stain positive, non-spore-forming, non-motile rods, 0.5 × 2 to 5 μm, and occur singly, in pairs, or in short chains, strictly anaerobic and catalase-negative. Colonies develop well on MRS agar plates supplemented with NAD under anaerobic conditions (1 mm in diameter), and not developed under aerobic conditions. Colonies on MRS+NAD agar plate are white, smooth, and approximately 1 to 2 mm in diameter after incubation for 2 days at 37°C. Homofermentative. No gas is produced from glucose. d- and l-lactic acid (in a ratio of 14:86) are produced as end products of glucose fermentation. Carbohydrate utilization profile and cellular fatty acid content are shown in Table S8 and S10. Cells grow well at 20°C to 37°C. The DNA G+C content is 34.9 mol%. The type strain is KimC2^T^ (= JCM 35347^T^ = DSM 114410^T^). This strain was isolated from a carpenter bee, *Xylocopa appendiculata circumvolans* (Japanese common name: *kimunekumabachi*), collected at Tokyo Kamiyouga Park located near the Tokyo University of Agriculture on July 7, 2017.

**(iii) Description of *Xylocopilactobacillus apicola* sp. nov.** *Xylocopilactobacillus apicola* (a.pi’co.la. L. fem. n. *apis*, a bee; L. suff. –*cola* [from L. masc. or fem. n. incola], inhabitant, dweller; N.L. masc. n. *apicola*, a dweller of bees). Cells are Gram-stain positive, non-spore-forming, non-motile rods, 0.5 × 2 to 5 μm, and occur singly, in pairs, or in short chains, strictly anaerobic and catalase-negative. Colonies develop well on MRS agar plates supplemented with NAD under anaerobic conditions (1 mm in diameter), and not developed under aerobic conditions. Colonies on MRS+NAD agar plate are white, smooth, and approximately 1 to 2 mm in diameter after incubation for 2 days at 37°C. Homofermentative. No gas is produced from glucose. d- and l-lactic acid (in a ratio of 17:83) are produced as end products of glucose fermentation. Carbohydrate utilization profile and cellular fatty acid content are shown in Table S8 and S10. Cells grow well at 30°C to 37°C. The DNA G+C content is 37.7 mol%. The type strain is XA3^T^ (= JCM 35348^T^ = DSM 114411^T^). This strain was isolated from a carpenter bee, *Xylocopa tranquebarorum* (Japanese common name: *taiwantakekumabachi*), collected at Tokyo Kinuta Park located near the Tokyo University of Agriculture on August 24, 2021.

**(iv) Description of *Lactobacillus xylocopicola* sp. nov.** *Lactobacillus xylocopicola* (xy.lo.co.pi’co.la. N.L. fem. n. *Xylocopa*, generic name of a bee; L. suff. –*cola* [from L. masc. or fem. n. incola], inhabitant, dweller; N.L. masc. n. *xylocopicola*, a dweller of *Xylocopa* bees). Cells are Gram-stain positive, non-spore-forming, non-motile rods, 0.5 × 2 to 5 μm, and occur singly, in pairs, or in short chains, facultatively anaerobic and catalase-negative. Colonies develop well on MRS agar plates under anaerobic conditions (1 mm in diameter), and not developed under aerobic conditions. Colonies on MRS agar are white, smooth, and approximately 1 to 2 mm in diameter after incubation for 2 days at 37°C. Homofermentative. No gas is produced from glucose. d- and l-lactic acid and acetic acid (in a ratio of 74:13:13) are produced as end products of glucose fermentation. Carbohydrate utilization profile and cellular fatty acid content are shown in Table S8 and S10. Cells grow well at 30°C to 37°C. The DNA G+C content is 42.9 mol%. The type strain is Kim32-2^T^ (= JCM 35343^T^ = DSM 108865^T^). This strain was isolated from a carpenter bee, *Xylocopa appendiculata circumvolans* (Japanese common name: *kimunekumabachi*), collected at Tokyo University of Agriculture on July 7, 2017.

**(v) Description of *Bombiscardovia nodaiensis* sp. nov.** Description of *Bombiscardovia nodaiensis* (no.dai.en’sis. N.L. fem. adj. *nodaiensis*, pertaining to the NODAI institute which is a Japanese common name of Tokyo University of Agriculture). Cells are Gram-stain positive, non-spore-forming, non-motile rods, 0.5 × 2 to 5 μm, and occur singly, in pairs, or in short chains, facultatively anaerobic and catalase-positive. Colonies develop well on MRS agar plates under anaerobic (1 mm in diameter), and slowly under aerobic (air, 1 mm in diameter) conditions. Colonies on MRS agar are white, smooth, and approximately 1 to 2 mm in diameter after incubation for 2 days at 37°C. No gas is produced from glucose. d- and l-lactic acid and acetic acid (in a ratio of 16:30:54) are produced as end products of glucose fermentation. Carbohydrate utilization profile and cellular fatty acid content are shown in Table S9 andS10. Cells grow well at 30°C to 37°C. The DNA G+C content is 57.6 mol%. The type strain is Kim37-2^T^ (= JCM 35346^T^ = DSM 114342^T^). This strain was isolated from a carpenter bee, *Xylocopa appendiculata circumvolans* (Japanese common name: *kimunekumabachi*), collected at Tokyo University of Agriculture on July 7, 2017.

**(vi) Description of *Bombiscardovia apis* sp. nov.** Description of *Bombiscardovia apis* (a’pis. L. gen. n. *apis*, of a bee). Cells are Gram-stain positive, non-spore-forming, non-motile rods, 0.5 × 2 to 5 μm, and occur singly, in pairs, or in short chains, facultatively anaerobic and catalase-negative. Colonies develop well on MRS agar plates under anaerobic (1 mm in diameter), and slowly under aerobic (air, 1 mm in diameter) conditions. Colonies on MRS agar are white, smooth, and approximately 1 to 2 mm in diameter after incubation for 3 days at 37°C. No gas is produced from glucose. d- and l-lactic acid and acetic acid (in a ratio of 14:29:57) are produced as end products of glucose fermentation. Carbohydrate utilization profile and cellular fatty acid content are shown in Table S9 and S10. Cells grow well at 30°C to 40°C. The DNA G+C content is 53.9 mol%. The type strain is KimH^T^ (= JCM 35345^T^ = DSM 114343^T^). This strain was isolated from a carpenter bee, *Xylocopa appendiculata circumvolans* (Japanese common name: *kimunekumabachi*), collected at Tokyo Kamiyouga Park located near the Tokyo University of Agriculture on July 7, 2017.

## MATERIALS AND METHODS

### Sample collection.

Samples were collected in Japan between 2016 and 2021. A total of 29 samples of *Xylocopa appendiculata circumvolans*, two samples of *Xylocopa flavifrons*, nine samples of *Xylocopa tranquebarorum*, 16 samples of honeybees, and 11 samples of bumble bees were collected at different places in Japan. The dates and the regions of sample collection are listed in Table S1. Bee species were identified by morphology (50). COI gene sequence similarities were also analyzed according to a previous literature (23, 24) using PCR primer coxF1: ATAATTTTTTTTATAGTTATAC and coxR1: GATGGGCTCATACAATAAATCCTA. The obtained COI gene sequences showed 99% to 100% identity to the morphologically identified species (*X. a. circumvolans*: EU861269; *X. flavifrons*: EU861286; *X. tranquebarorum*: LC257680).

### DNA extraction and gut microbiome analysis.

Bee samples were collected in sterilized tubes, cooled at 4°C, and the body surface was rinsed in 70% ethanol to reduce the effect of environmental microbes. By using forceps and scissors for dissection, bee bodies were dissected, and the entire gut were removed and placed on petri dish. Microbial DNA was extracted with standard protocol using a bead beater instrument (Multi-Beads Shocker, Yasui Kikai, Japan). Removed gut samples were homogenized and resuspended in lysis buffer (50 mM Tris-HCl, 1 mM EDTA, containing lysozyme and proteinase K) and incubated for 30 min at 37°C and 10 min at 55°C. After adding 1% SDS, TE-saturated phenol was added, and the solutions were processed with a bead beater for 40 s twice. The aqueous solution were treated by phenol:chloroform to remove proteins, and the genome DNA was extracted by ethanol precipitation. The V1 to V3 region of the 16S rRNA gene was amplified using the 27F-Illumina forward primer (5′-TCGTCGGCAGCGTCAGATGTGTATAAGAGACAGAGAGTTTGATCCTGGCTCAG-3′) and the 518R-Illumina reverse primer (5′-GTCTCGTGGGCTCGGAGATGTGTATAAGAGACAGATTACCGCGGCTGCTGG-3′) according to the manufacturer’s instructions. The 16S rRNA gene amplicon analysis was performed using Illumina Miseq.

### Sequence analysis.

The 16S rRNA gene sequencing data of the 5′ forward reads were analyzed by QIIME2 software ver. 2019.10 (51). The DADA2 plugin was used for primer trimming, filtering low quality sequences, denoising, removing chimeric reads, and ASV calling. We visualized quality scores and trimmed the reads when quality scores dropped below 30. Quality parameters were listed in Data set S2. Sequencing reads were truncated at 260 bp. Silva database version 132 was used to assign taxonomy in QIIME2 pipeline. Sequences with chloroplast and mitochondrion assignments were removed. Assignment of bacterial species corresponding to each ASV was performed by BLASTn searches against the NCBI 16S rRNA database (last modified on November 7, 2022) to obtain the BLAST top hit. ASVs that showed BLASTN >97% similarity were manually extracted and assigned taxon names from the BLAST top hits and the relative abundance toward total reads of each sample was calculated. We visualized overall differences in microbial communities across sample types with PCoA applied to weighted UniFrac distance matrices of log-transformed abundance data by QIIME2. Analysis of similarities (ANOSIM) and permutational multivariate analysis of variance (PERMANOVA) were used for statistical testing of group similarities by QIIME2.

### Isolation and characterization of strains.

Gut samples were collected using sterile scalpel and forceps and cultivated under anaerobic conditions on MRS agar (Difco) containing 15 g/L agar. After isolation of the bacterial colonies, the isolated strains were maintained in MRS broth. Many colonies were obtained, and the 16S rRNA genes were amplified using the primers 27F (5′-AGAGTTTGATCCTGGCTCAG-3′) and 1525R (5′-AAAGGAGGTGATCCAGCC-3′) primers for randomly selected colonies. Colonies were sometimes difficult to isolate pure single colony due to the co-existence of contaminant strains, which was found by checking the sequence raw chart. KimC2 was developed a pure single colony by adding cultured supernatants of other bacteria. The strain XA3 was developed a pure single colony by adding 3 mM NAD^+^ (Sigma, USA) in MRS agar plate. Phenotypic and biochemical tests were performed as described previously (32, 33, 49, 52). Briefly, cell morphology, Gram stain, and several biochemical studies were examined on cells grown in MRS broth at 37°C with the addition of 3 mM NAD^+^ if necessary. The morphology of the cells grown on MRS agar plates for 48 h at 37°C under anaerobic conditions was observed under a microscope (H550L, Nikon, Japan). Catalase was detected by placing one drop of 3% (wt/vol) H_2_O_2_ onto the colonies on the MRS agar plate (49). Motility was tested in MRS soft agar. Acid production from carbohydrates and enzyme activity patterns of the novel species and all reference strains were examined using the API 50CHL, with the addition of 3 mM NAD^+^ in the base medium if necessary, in triplicate according to the manufacturer’s instructions. Determining fermentation products and carbohydrate utilization were used the modified MRS medium (without 0.5% sodium acetate) containing 1% (wt/vol) carbohydrates, 1% proteose peptone, 0.2% beef extract, 0.5% yeast extract, 0.2% ammonium citrate, 0.02% MgCl_2_, 0.2% K_2_HPO_4_, and 0.005% MnSO_4_, pH 7.0, with the addition of 3 mM NAD^+^ if necessary. Production of d- and l-lactic acid from d-glucose was confirmed by performing HPLC analysis with a separation column for optical isomers (CRS10W column; Mitsubishi Chemical, Japan) (52). Acetic acid production was also detected under the same HPLC condition. Fatty acid methyl esters were prepared from cells grown on MRS agar that had been incubated for 48 h at 30°C. Various processes, including methylation and extraction, among others, were undertaken as described previously (52, 53). Cellular fatty acid profiles were determined by following version 6.2B of the Sherlock Microbial Identification System (MIDI) and using version 6.21 of the TSBA6 database. Gas production was detected using Durham tubes in modified MRS medium of static culture conditions at 37°C. Salt tolerance was examined in MRS broth containing 0% to 10% (wt/vol) NaCl at 37°C. nicotinamide (NM), nicotinate (NA), Nicotinate adenine dinucleotide (NaAD), NAD(H), and NADP(H) were purchased from Sigma-Aldrich (St. Louis, USA) or Wako (Osaka, Japan). The full-length 16S rRNA genes were amplified, sequenced, and constructed phylogenies together with related taxa. Reference sequences for the type strains of Lactobacillaceae and Bifidobacteriaceae species were downloaded from NCBI RefSeq database. The 16S rRNA gene sequences were aligned using ClustalX, and phylogenetic analyses were inferred in MEGA X (54) by using the maximum-likelihood method under the best-fit model (GTR+G+I), with 1000 bootstrap replications and complete deletion of gaps option.

### Genome sequencing.

Whole-genome sequencing was performed for the five candidate novel species. High-quality genomic DNA was extracted from cultured bacteria using standard protocols, and the genome was sequenced with the PacBio Sequel or RSII sequencing platform at Macrogen (Macrogen, Seoul, Korea). Sequence reads were assembled using CANU version 1.0.6 with default parameters under the Maser platform (55). The genome sequence data were annotated using DFAST v1.2.15 and the predicted protein sequences were functionally annotated using the BlastKOALA online tool, which is an international annotation tool of Kyoto encyclopedia of genes and genomes (KEGG) (56). To categorize the function of genes, we analyzed the genome information by COGs function categories (57). Reverse-position-specific BLAST (RPS-BLAST) was performed against the COG database and classified by cdd2cog.pl script (58). To confirm the taxonomic position of the *Xylocopa*-specific core species (KimC2 and XA3), a core genome phylogenetic analysis was performed using BPGA v1.3 (59). The BPGA tool searched for core genes from the genomes of the selected type strains of Lactobacillaceae (35). The concatenated 127 single-copy core genes were aligned by MUSCLE and the phylogenetic tree was constructed by the maximum likelihood method using the best model (LG + I + G + F) in RAxML-NG (60) (2019 RaxML) and visualized by iTOL (https://itol.embl.de/). We generated graphic illustrations of the genome map using CGView (61). The chromosome sequences of each genome were independently aligned against that of the reference genome using PROmer implemented in MUMmer4 package (62). The nucleotide sequences of the reference strains were obtained from the NCBI database. The digital DNA-DNA hybridization (dDDH) values were determined using Genome-to-Genome Distance Calculator (GGDC) version 3.0 (63), and ANI values among strains and reference genomes were calculated using the ANI calculator (64). The average amino acid identity (AAI) values were calculated using AAI calculator (42).

### Data availability.

All the novel strains have been deposited in Japan collection of microorganisms (JCM) and German Collection of Microorganisms (DSMZ). All sequence data and metadata are accessible at NCBI under BioProject numbers PRJDB13358-PRJDB13360. 16S rRNA gene sequences of the candidate novel species were under accession numbers LC726283-LC726297. The complete genome sequences of the five novel strains KimC2, XA3, Kim32-2, Kim37-2, Kim37-2, and KimH, and a plasmid of Kim37-2 have been deposited in the GenBank database under accession number AP026801, AP026802, AP20803, AP026798, AP026800, and AP026799, respectively. All raw reads data from these five strains are accessible at DDBJ Sequence Read Archive (DRA) database under the accession numbers DRA013792 and DRA013793. All sequenced data obtained from insect gut microbiome have been deposited in the DRA database under the accession number DRA015622.
