# Supplementary material for: Symbiosis of Carpenter Bees with Uncharacterized Lactic Acid Bacteria Showing NAD Auxotrophy
Source: Microbiol Spectr. 2023 Jun 22;11(4):e00782-23. doi: 10.1128/spectrum.00782-23 (PMC10433979; doi:10.1128/spectrum.00782-23)
Supplement: Supplemental file 1 — Supplemental material. Download spectrum.00782-23-s0001.pdf, PDF file, 2.6 MB [file spectrum.00782-23-s0001.pdf]

Supplemental materials for

Symbiosis of carpenter bees with uncharacterized lactic acid bacteria showing NAD auxotrophy

Shinji Kawasaki[a#], Kaori Ozawa[a], Tatsunori Mori[a], Arisa Yamamoto[a], Midoriko Ito[a], Moriya Ohkuma[b], Mitsuo Sakamoto[b], Minenosuke Matsutani[c]

[a]Department of Molecular Microbiology, Tokyo University of Agriculture, 1-1-1 Sakuragaoka, Setagaya-ku, Tokyo 156-8502, Japan

[b]Microbe Division/Japan Collection of Microorganisms, RIKEN BioResource Research Center, Tsukuba, Ibaraki 305-0074, Japan

[c]NODAI Genome Research Center, Research Institute, Tokyo University of Agriculture, Setagaya-ku, Sakuragaoka 1-1-1, Tokyo 156-8502, Japan

#Corresponding author : Email: kawashin@nodai.ac.jp

Table of contents

FIG S1 Pictures of the *Xylocopa* carpenter bees collected in Japan.

Fig S2 Phylogenetic tree based on 16S rRNA gene sequences of Lactobacillaceae.

Fig S3 Phylogenetic tree based on 16S rRNA gene sequences of Bifidobacteriaceae.

FIG S4 Comparison of number of genes associated with functional categories classified by Clusters of Orthologous Groups (COGs) function categories.

FIG S5 Cell morphology of novel strains.

TABLE S1 List of bee samples with sampling area information.

TABLE S2 The average nucleotide identity (ANI, top, bold type) and the digital DNA-DNA hybridization (dDDH, bottom, normal type) values (%) between the isolates (KimC2 and XA3) and their related taxa.

TABLE S3 The average nucleotide identity (ANI, top, bold type) and the digital DNA-DNA hybridization (dDDH, bottom, normal type) values (%) between an isolate (Kim32-2) and its related taxa.

TABLE S4 The average nucleotide identity (ANI, top, bold type) and the digital DNA-DNA hybridization (dDDH, bottom, normal type) values (%) between the isolates (Kim37-2 and KimH)) and their related taxa.

TABLE S5 Differential phenotypic characteristics between isolates and closely related species.

TABLE S6 Differential phenotypic characteristics between isolates and closely related species.

TABLE S7 Cellular fatty acid content (%) of isolates from *Xylocopa* species.

TABLE S8 Summary of whole genome sequence obtained from the Pacific Bioscience (PacBio) and assembly.

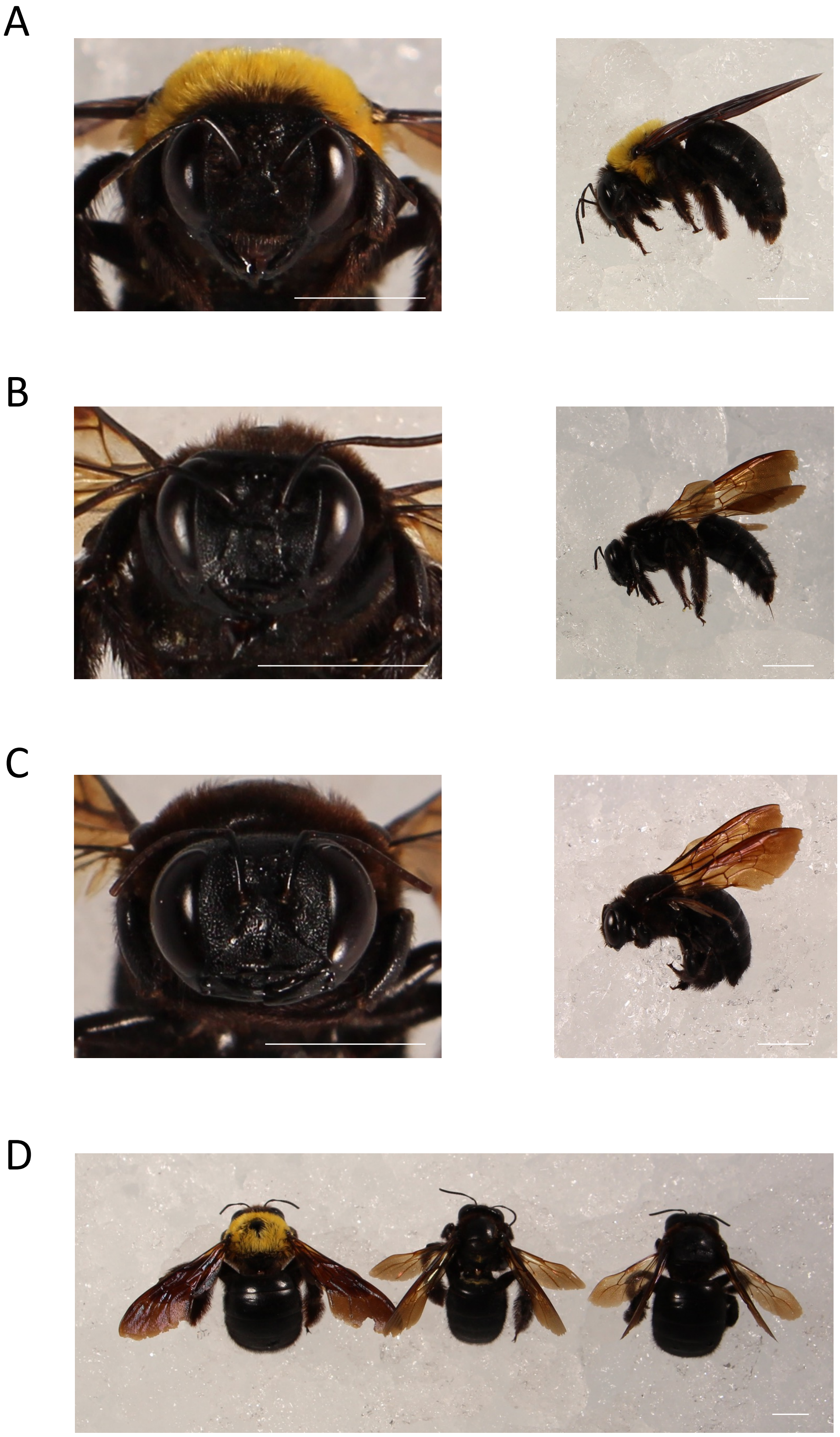

FIG S1 Pictures of the *Xylocopa* carpenter bees collected in Japan. A. *Xylocopa appendiculate circumvolans;* B. *X. tranquebarorum;* C. *X. flavifrons.* D. Comparison of three *Xylocopa* species. Scale bar: 0.5 cm.

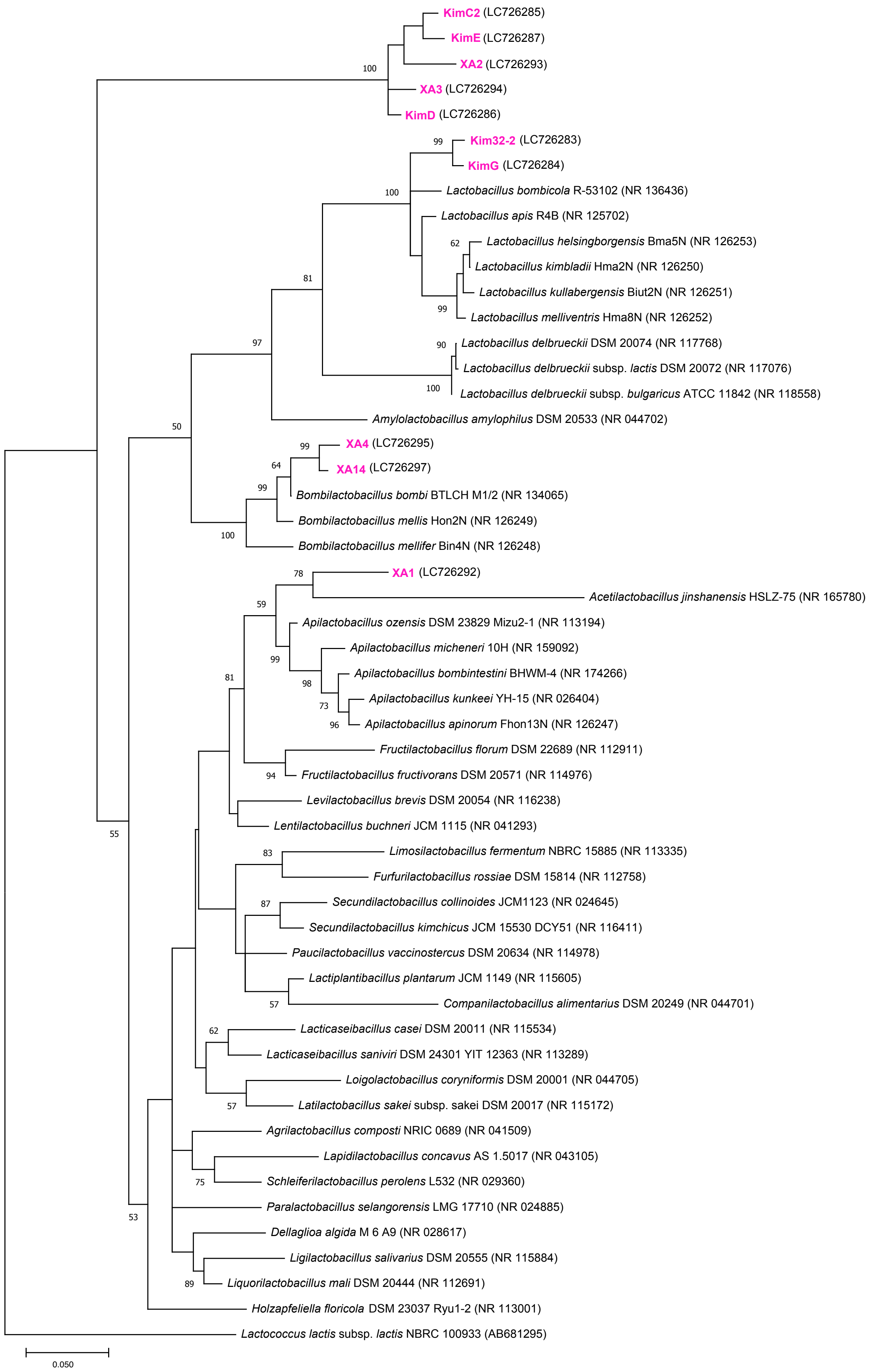

Fig S2 Phylogenetic tree based on 16S rRNA gene sequences of Lactobacillaceae. The tree was constructed using the maximum-likelihood method inferred by MEGAX using the best model (GT + G+ I). Bootstrap support values were calculated from 1000 replicates, and values above 50% are labeled. *Lactococcus lactis* NBRC100933 was used as an outgroup. Candidates of novel species of isolates are shown in pink font. Bar, 0.05 substitutions per nucleotide position.

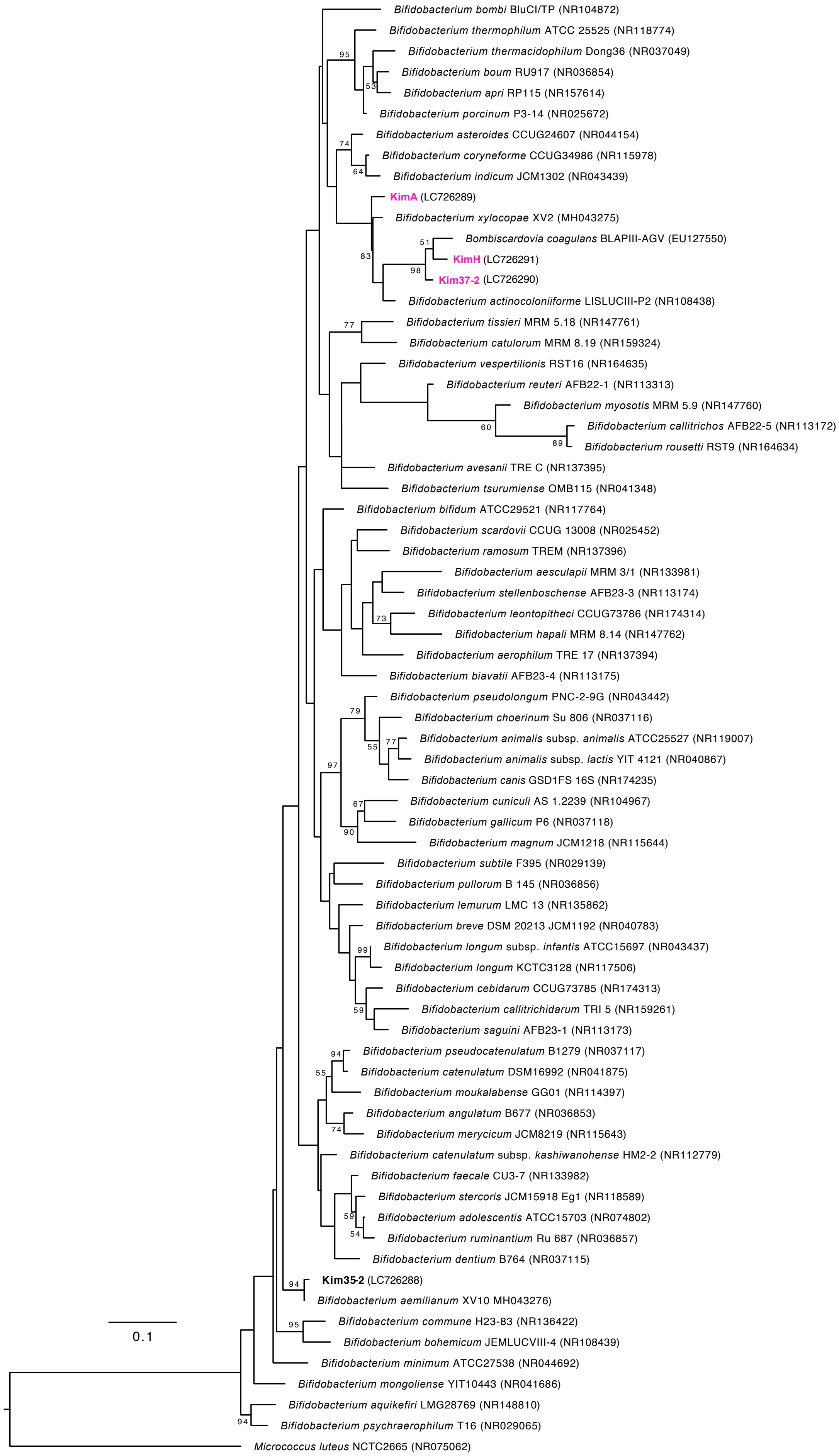

Fig S3 Phylogenetic tree based on 16S rRNA gene sequences of Bifidobacteriaceae. The tree was constructed using the maximum-likelihood method inferred by MEGAX using the best model (GT + G+ I). Bootstrap support values were calculated from 1000 replicates, and values above 50% are labeled. Candidate of novel species of isolates are shown in pink font. Bar, 0.1 substitutions per nucleotide position.

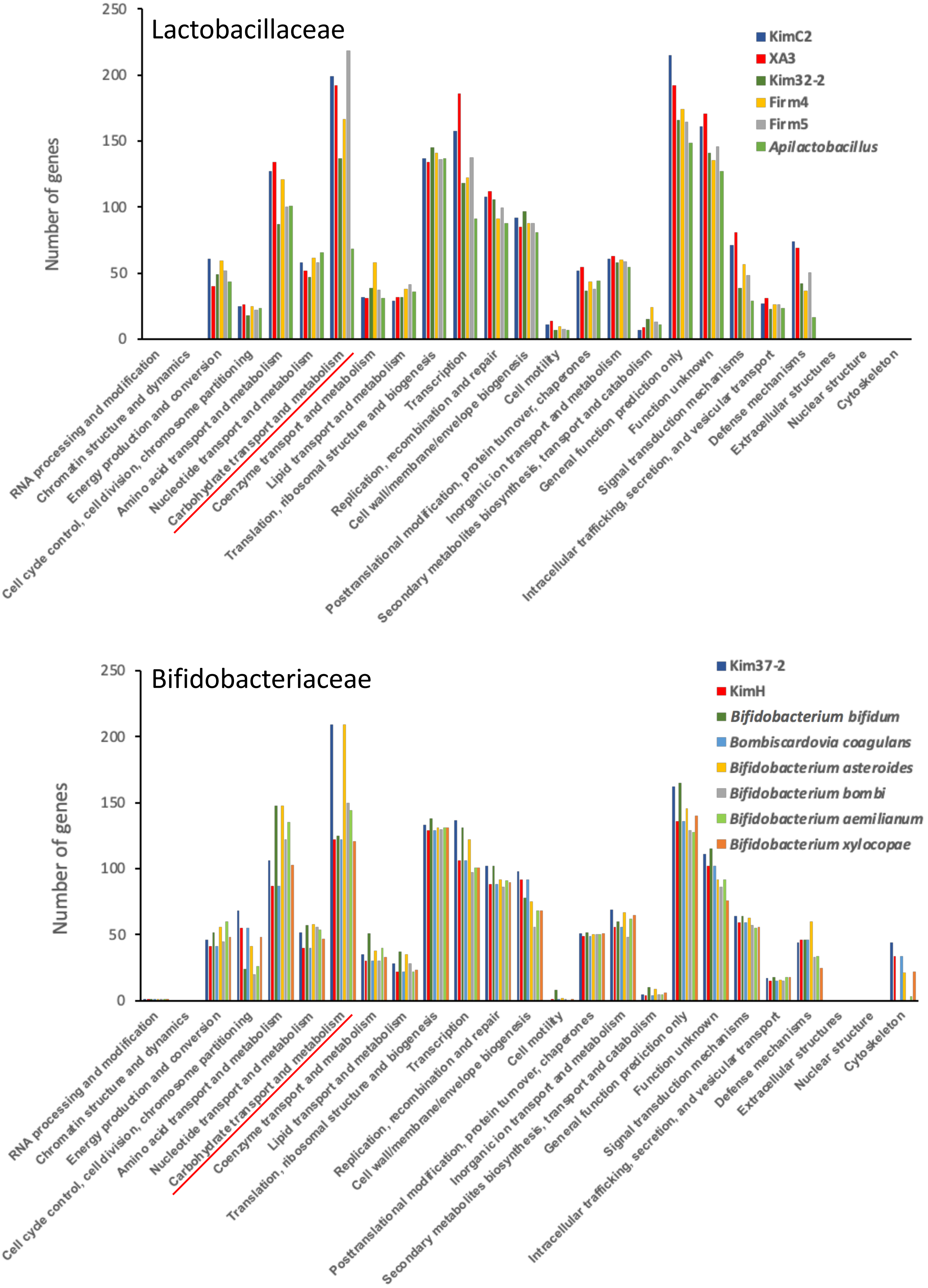

FIG S4 Comparison of number of genes associated with functional categories classified by Clusters of Orthologous Groups (COGs) function categories. Red line indicates category to which a large number of genes belong.

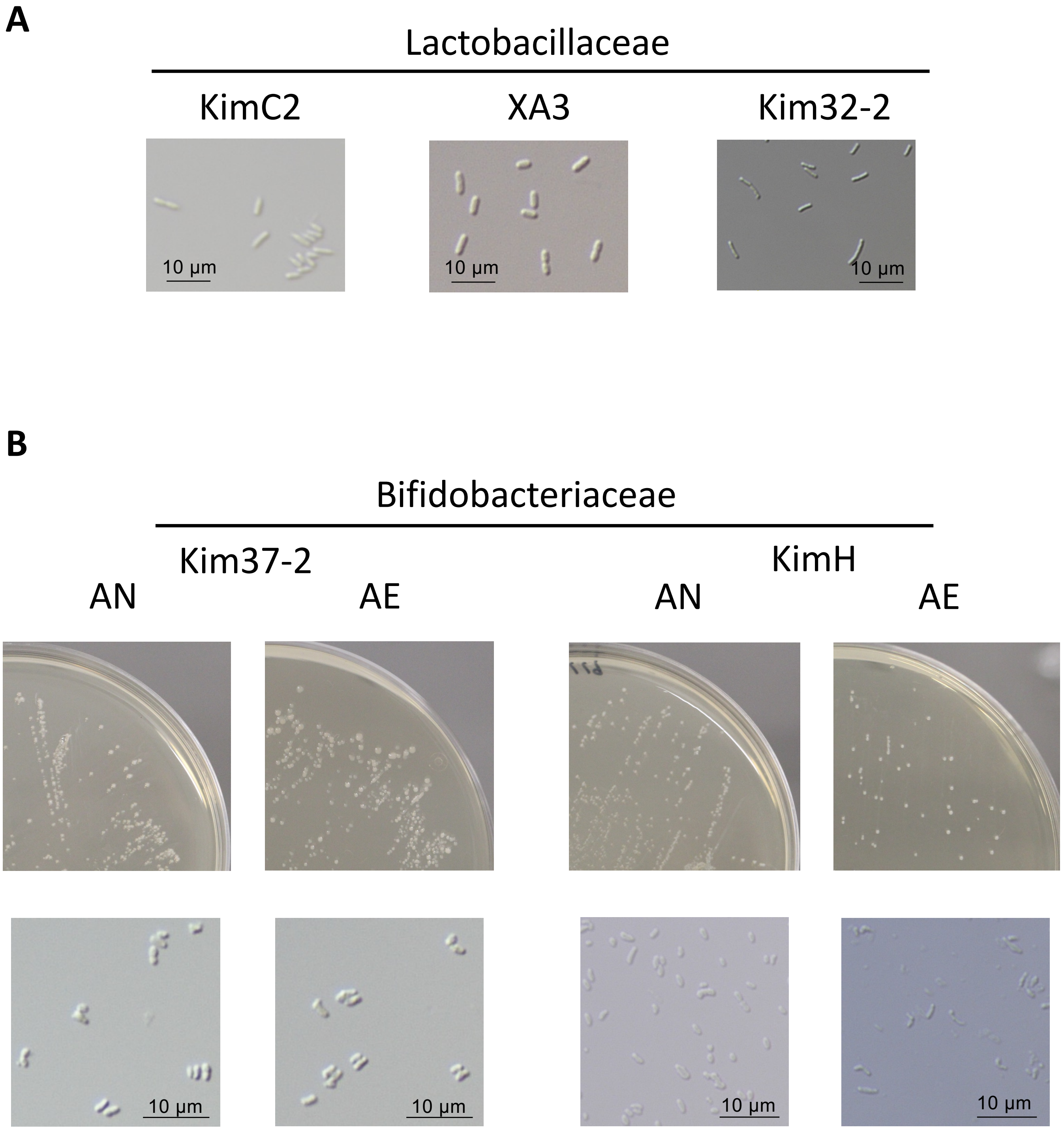

FIG S5 Cell morphology of novel strains. A. Cell morphology of the strains KimC2, XA3, and Kim32-2 based on differential interference microscopy. B. Colony development and cell morphology of the strains Kim37-2 and KimH grown under strict anaerobic (N_2 100%, AN) and aerobic (air 100%, AE) conditions.

TABLE S1 List of bee samples with sampling area information.

Bee ID	Species	Collection year	Collection month	Sampling prefecture	Sampling area	Latitude	Longitude
K1	*Xylocopa appendiculate circumvolans*	2016	6	Aichi	Irokaneyama-park	35.186465	137.055549
K2	*Xylocopa appendiculate circumvolans*	2016	7	Tokyo	Planting fireld in the area of Tokyo Univiversity of Agricultur	35.641211	139.632157
K3	*Xylocopa appendiculate circumvolans*	2016	7	Tokyo	Planting fireld in the area of Tokyo Univiversity of Agricultur	35.641211	139.632157
K4	*Xylocopa appendiculate circumvolans*	2016	7	Tokyo	Planting fireld in the area of Tokyo Univiversity of Agricultur	35.641211	139.632157
K5	*Xylocopa appendiculate circumvolans*	2016	7	Tokyo	Planting fireld in the area of Tokyo Univiversity of Agricultur	35.641211	139.632157
K6	*Xylocopa appendiculate circumvolans*	2016	9	Tokyo	Shikine island	34.327047	139.210656
K7	*Xylocopa appendiculate circumvolans*	2016	9	Tokyo	Shikine island	34.327047	139.210656
K8	*Xylocopa appendiculate circumvolans*	2017	7	Tokyo	Kamiyoga-park near the area of Tokyo Univiversity of Agric	35.638531	139.628958
K9	*Xylocopa appendiculate circumvolans*	2017	8	Saitama	Ageo city	35.972496	139.602429
K10	*Xylocopa appendiculate circumvolans*	2017	8	Ibaraki	Koga city	36.198391	139.831409
K11	*Xylocopa appendiculate circumvolans*	2018	5	Tokyo	Kamiyoga-park near the area of Tokyo Univiversity of Agric	35.638531	139.628958
K12	*Xylocopa appendiculate circumvolans*	2018	5	Tokyo	Kamiyoga-park near the area of Tokyo Univiversity of Agric	35.638531	139.628958
K13	*Xylocopa appendiculate circumvolans*	2019	4	Osaka	Sakai city	34.572458	135.530176
K14	*Xylocopa appendiculate circumvolans*	2019	4	Tokyo	Kinuta-park near the area of Tokyo Univiversity of Agricultu	35.631164	139.624533
K15	*Xylocopa appendiculate circumvolans*	2019	4	Osaka	Sakai city	34.572458	135.530176
K16	*Xylocopa appendiculate circumvolans*	2019	7	Aichi	Meijyo-park	35.189211	136.902255
K17	*Xylocopa appendiculate circumvolans*	2019	7	Aichi	Meijyo-park	35.189211	136.902255
K18	*Xylocopa appendiculate circumvolans*	2019	6	Tokyo	Kinuta-park near the area of Tokyo Univiversity of Agricultu	35.631164	139.624533
K19	*Xylocopa appendiculate circumvolans*	2019	6	Tokyo	Kinuta-park near the area of Tokyo Univiversity of Agricultu	35.631164	139.624533
K20	*Xylocopa appendiculate circumvolans*	2019	6	Tokyo	Kinuta-park near the area of Tokyo Univiversity of Agricultu	35.631164	139.624533
K21	*Xylocopa appendiculate circumvolans*	2019	9	Tokyo	Kinuta-park near the area of Tokyo Univiversity of Agricultu	35.631164	139.624533
K22	*Xylocopa appendiculate circumvolans*	2019	9	Tokyo	Kinuta-park near the area of Tokyo Univiversity of Agricultu	35.631164	139.624533
K23	*Xylocopa appendiculate circumvolans*	2020	8	Tokyo	Kinuta-park near the area of Tokyo Univiversity of Agricultu	35.631164	139.624533
K24	*Xylocopa appendiculate circumvolans*	2020	8	Tokyo	Kinuta-park near the area of Tokyo Univiversity of Agricultu	35.631164	139.624533
K25	*Xylocopa appendiculate circumvolans*	2020	8	Tokyo	Kinuta-park near the area of Tokyo Univiversity of Agricultu	35.631164	139.624533
K26	*Xylocopa appendiculate circumvolans*	2020	8	Tokyo	Kinuta-park near the area of Tokyo Univiversity of Agricultu	35.631164	139.624533
K27	*Xylocopa appendiculate circumvolans*	2020	8	Tokyo	Kinuta-park near the area of Tokyo Univiversity of Agricultu	35.631164	139.624533
K28	*Xylocopa appendiculate circumvolans*	2020	10	Tokyo	Kinuta-park near the area of Tokyo Univiversity of Agricultu	35.631164	139.624533
K29	*Xylocopa appendiculate circumvolans*	2020	10	Tokyo	Kinuta-park near the area of Tokyo Univiversity of Agricultu	35.631164	139.624533
T1	*Xylocopa tranquebarorum*	2016	6	Aichi	Irokaneyama-park	35.186465	137.055549
T2	*Xylocopa tranquebarorum*	2016	6	Aichi	Irokaneyama-park	35.186465	137.055549
T3	*Xylocopa tranquebarorum*	2019	7	Aichi	Meijyo-park	35.189211	136.902255
T4	*Xylocopa tranquebarorum*	2019	7	Aichi	Meijyo-park	35.189211	136.902255
T5	*Xylocopa tranquebarorum*	2019	7	Aichi	Meijyo-park	35.189211	136.902255
T6	*Xylocopa tranquebarorum*	2016	6	Aichi	Irokaneyama-park	35.186465	137.055549
T7	*Xylocopa tranquebarorum*	2016	6	Aichi	Irokaneyama-park	35.186465	137.055549
T8	*Xylocopa tranquebarorum*	2016	6	Aichi	Nagakute city	35.170251	137.036995
T9	*Xylocopa tranquebarorum*	2020	8	Tokyo	Kinuta-park near the area of Tokyo Univiversity of Agricultu	35.631164	139.624533
O1	*Xylocopa flavifrons*	2018	10	Okinawa	Kunigami Oku	26.836951	128.287758
O1	*Xylocopa flavifrons*	2018	10	Okinawa	Kunigami Oku	26.836951	128.287758
H1	*Apis mellifera*	2016	8	Tokyo	Baji near the area of Tokyo Univiversity of Agriculture	35.636949	139.633163
H2	*Apis mellifera*	2016	11	Tokyo	Baji near the area of Tokyo Univiversity of Agriculture	35.636949	139.633163
H3	*Apis mellifera*	2016	8	Tokyo	Baji near the area of Tokyo Univiversity of Agriculture	35.636949	139.633163
H4	*Apis mellifera*	2016	12	Tokyo	Baji near the area of Tokyo Univiversity of Agriculture	35.636949	139.633163
H5	*Apis mellifera*	2016	12	Tokyo	Baji near the area of Tokyo Univiversity of Agriculture	35.636949	139.633163
H6	*Apis mellifera*	2016	12	Tokyo	Baji near the area of Tokyo Univiversity of Agriculture	35.636949	139.633163
H7	*Apis mellifera*	2016	12	Tokyo	Baji near the area of Tokyo Univiversity of Agriculture	35.636949	139.633163
H8	*Apis mellifera*	2016	12	Tokyo	Baji near the area of Tokyo Univiversity of Agriculture	35.636949	139.633163
H9	*Apis mellifera*	2016	12	Tokyo	Baji near the area of Tokyo Univiversity of Agriculture	35.636949	139.633163
H10	*Apis mellifera*	2016	12	Tokyo	Baji near the area of Tokyo Univiversity of Agriculture	35.636949	139.633163
H11	*Apis mellifera*	2016	6	Aichi	Irokaneyama-park	35.186465	137.055549
H12	*Apis mellifera*	2016	6	Aichi	Irokaneyama-park	35.186465	137.055549
H13	*Apis mellifera*	2016	7	Gunma	Oze park	36.874873	139.305438
H14	*Apis mellifera*	2016	7	Tokyo	Baji near the area of Tokyo Univiversity of Agriculture	35.636949	139.633163
H15	*Apis mellifera*	2017	5	Hyogo	Awaji island	34.346517	134.839661
H16	*Apis mellifera*	2020	8	Tokyo	Kinuta-park near the area of Tokyo Univiversity of Agricultu	35.631164	139.624533
M1	*Bombus terrestris*	2016	6	Tokyo	Baji near the area of Tokyo Univiversity of Agriculture	35.636949	139.633163
M2	*Bombus terrestris*	2016	6	Tokyo	Baji near the area of Tokyo Univiversity of Agriculture	35.636949	139.633163
M3	*Bombus terrestris*	2016	7	Gunma	Oze park	36.874873	139.305438
M5	*Bombus terrestris*	2016	9	Nagano	Nonomi park	37.026166	138.522315
M6	*Bombus terrestris*	2016	9	Nagano	Nonomi park	37.026166	138.522315
M7	*Bombus hypocrita*	2016	6	Hokkaido	Asahidake	43.663608	142.854131
M8	*Bombus hypocrita*	2016	6	Hokkaido	Asahidake	43.663608	142.854131
M9	*Bombus hypocrita*	2016	6	Hokkaido	Furano city	43.329643	142.440334
M10	*Bombus terrestris*	2016	6	Hokkaido	Asahidake	43.663608	142.854131
M11	*Bombus ardens ardens*	2016	9	Tokyo	Baji near the area of Tokyo Univiversity of Agriculture	35.636949	139.633163
M12	*Bombus ardens ardens*	2016	9	Tokyo	Baji near the area of Tokyo Univiversity of Agriculture	35.636949	139.633163

TABLE S2 The average nucleotide identity (ANI, top, bold type) and the digital DNA-DNA hybridization (dDDH, bottom, normal type) values (%) between the isolates (KimC2 and XA3) and their related taxa.

	1	2	3	4	5
**1. KimC2**	–	**75.0**	**68.5**	**66.0**	**66.3**
**2. XA3**	20.0	–	**68.6**	**65.4**	**65.6**
**3. *Bombilactobacillus bombi* BI-2.5**	23.1	21.1	–	**67.7**	**67.0**
**4. *Lactobacillus apis* ESL0185**	23.6	23.6	22.1	–	**65.6**
**5. *Lacticaseibacillus saniviri* JCM 17471^T**	25.8	31.0	27.7	25.6	–

Accession number of the whole genome sequence are KimC2^T (AP026789), XA3^T (AP026798), *Bombilactobacillus bombi* BI-2.5 (CP031513), *Lactobacillus apis* ESL0185 (CP029476), and *Lacticaseibacillus saniviri* JCM 17471^T (NZ_JQCE01000000).

TABLE S3 The average nucleotide identity (ANI, top, bold type) and the digital DNA-DNA hybridization (dDDH, bottom, normal type) values (%) between an isolate (Kim32-2) and its related taxa.

	1	2	3
**1. Kim32-2**	–	**73.7**	**73.9**
**2. *Lactobacillus bombicola* H70-3**	18.2	–	**75.7**
**3. *Lactobacillus apis* ESL0185**	18.9	19.3	–

Accession number of the whole genome sequence are Kim32-2^T (AP026800), *Lactobacillus bombicola* H70-3 (NZ_NPNG01000000), and *Lactobacillus apis* ESL0185 (CP029476).

TABLE S4 The average nucleotide identity (ANI, top, bold type) and the digital DNA-DNA hybridization (dDDH, bottom, normal type) values (%) between the isolates (Kim37-2 and KimH)) and their related taxa.

	1	2	3
**1. Kim37-2**	–	**76.4**	**71.4**
**2. KimH**	22.1	–	**71.9**
**3. *Bombiscardovia coagulans* DSM 22924^T**	22.1	17.6	–

Accession number of the whole genome sequence are Kim37-2^T (AP026802), KimH (AP026803), and *Bomscardovia coagulans* DSM 22924^T (NZ_MWWS00000000).

TABLE S5 The average amino acid identity (AAI) value (%) between the isolates (KimC2 and XA3) and their related taxa.

	1	2	3	4	5
**1. KimC2**	–				
**2. XA3**	73.8	–			
**3.** ***Bombilactobacillus bombi*** **BI-2.5**	50.9	51.3	–		
**4.** ***Lactobacillus apis*** **ESL0185**	48.2	48.2	52.0	–	
**5.** ***Lacticaseibacillus saniviri*** **JCM 17471^T**	48.5	48.6	52.2	49.7	–

Accession number of the whole genome sequence are KimC2^T (AP026789), XA3^T (AP026798), *Bombilactobacillus bombi* BI-2.5 (CP031513), *Lactobacillus apis* ESL0185 (CP029476), and *Lacticaseibacillus saniviri* JCM 17471^T (NZ_JQCE01000000).

TABLE S6 The average amino acid identity (AAI) value (%) between an isolate (Kim32-2) and its related taxa.

	1	2	3
**1. Kim32-2**	–		
**2.** ***Lactobacillus bombicola*** **H70-3**	75.2	–	
**3.** ***Lactobacillus apis*** **ESL0185**	76.9	76.8	–

Accession number of the whole genome sequence are Kim32-2^T (AP026800), *Lactobacillus bombicola* H70-3 (NZ_NPNG01000000), and *Lactobacillus apis* ESL0185 (CP029476).

TABLE S7 The average amino acid identity (AAI) value (%) between the isolates (Kim37-2 and KimH)) and their related taxa.

**1. Kim37-2**	–		
**2. KimH**	77.6	–	
**3.** ***Bombiscardovia coagulans*** **DSM 22924^T**	72.7	74.4	–

Accession number of the whole genome sequence are Kim37-2^T (AP026802), KimH (AP026803), and *Bomscardovia coagulans* DSM 22924^T (NZ_MWWS00000000).

TABLE S8 Differential phenotypic characteristics between isolates and closely related species. KimC2 (JCM 35347^T), XA3 (JCM 35348^T), Kim32-2 (JCM 35343^T), *Lactobacillus bombicola* (LMG 28288^T), *Bombilactobacillus bombi* (DSM 26517^T). +, Positive; –, Negative; w, weakly positive; NT, not tested. For KimC2, acid is produced from D-glucose, D-fructose, D-mannose, *N*-acetylglucosamine, D-mannitol, L-rhamnose, arbutin, salicin, D-cellobiose, D-sucrose, D-trehalose, gentiobiose, D-tagatose. No acid is produced from glycerol, erythritol, D-arabinose, esculin, D-galactose, amygdalin, L-sorbose, D-xylose, L-xylose, D-adonitol, dulcitol, inositol, D-sorbitol, methyl-D-xylopyranoside, methyl-D-mannopyranoside, methyl-D-glucopyranoside, D-maltose, D-lactose, D-melibiose, inulin, D-melezitose, D-raffinose, starch, glycogen, xylitol, D-turanose, D-lyxose, D-fucose, L-fucose, D-arabitol, L-arabitol, gluconate, D-ribose, L-arabinose, 2-keto gluconate, and 5-keto gluconate. For XA3, acid is produced from D-glucose, D-fructose, D-mannose, *N*-acetylglucosamine,salicin, D-cellobiose, D-sucrose, gentiobiose. No acid is produced from glycerol, erythritol, D-arabinose, esculin, D-galactose, L-sorbose, L-rhamnose, D-xylose, L-xylose, D-adonitol, dulcitol, inositol, D-sorbitol, methyl-D-xylopyranoside, methyl-D-mannopyranoside, methyl-D-glucopyranoside, D-maltose, D-lactose, D-melibiose, inulin, D-melezitose, D-raffinose, starch, glycogen, xylitol, D-turanose, D-lyxose, D-fucose, L-fucose, D-arabitol, L-arabitol, gluconate, D-ribose, D-trehalose, L-arabinose, 2-keto gluconate, 5-keto gluconate, D-mannitol, D-tagatose, and arbutin. For Kim32-2, acid is produced from D-glucose, D-fructose, D-mannose, D-mannitol, *N*-acetylglucosamine, salicin, D-cellobiose, gentiobiose, D-tagatose. No acid is produced from glycerol, erythritol, D-arabinose, esculin, D-galactose, amygdalin, L-sorbose, L-rhamnose, D-xylose, L-xylose , D-adonitol, dulcitol, inositol, D-sorbitol, methyl-D-xylopyranoside, methyl-D-mannopyranoside, methyl-D-glucopyranoside, D-maltose, D-lactose, D-melibiose, D-sucrose, inulin, D-melezitose, D-raffinose, starch, glycogen, xylitol, D-turanose, D-lyxose, D-fucose, L-fucose, D-arabitol, L-arabitol, gluconate, D-ribose, D-trehalose, L-arabinose, 2-keto gluconate, and 5-keto gluconate.

	**KimC2**	**XA3**	**Kim32-2**	***Lactobacillus bombicola***	***Bombilactobacillus bombi***
**Lactic acid isomers**	**DL**	**DL**	**DL**	**D**	**DL**
**Spore formation**	**–**	**–**	**–**	**–**	**–**
**Motility**	**–**	**–**	**–**	**–**	**–**
**Homo or hetero**	**homo**	**homo**	**homo**	**homo**	**homo**
**Nitrate reduction**	**-**	**-**	**-**	**NT**	**–**
**Growth temperature**	**10-40**	**30-40**	**25-40**	**25-37**	**20-47**
**GC content**	**34.9**	**37.7**	**42.9**	**34.6**	**34.7**
**Genome size Mbp**	**2.27**	**2.31**	**1.72**	**1.64**	**1.84**
Utilization of:					
**L-Arabinose**	**–**	**–**	**–**	**+**	**+**
**D-Xylose**	**–**	**–**	**–**	**–**	**+**
**D-Glucose**	**+**	**+**	**+**	**+**	**+**
**D-Fructose**	**+**	**+**	**+**	**+**	**+**
**D-Mannose**	**+**	**+**	**+**	**+**	**+**
**L-Rhamnose**	**+**	**–**	**–**	**–**	**W**
**D-Mannitol**	**+**	**–**	**+**	**–**	**–**
**N-Acethyl Glucosamine**	**+**	**+**	**+**	**+**	**W**
**Amygdalin**	**–**	**–**	**–**	**+**	**+**
**Arbutin**	**+**	**–**	**–**	**+**	**+**
**Salicin**	**+**	**+**	**+**	**+**	**+**
**D-Cellobiose**	**+**	**+**	**+**	**+**	**+**
**D-Melibiose**	**–**	**–**	**–**	**–**	**+**
**D-Sucrose**	**+**	**+**	**–**	**–**	**+**
**D-Trehalose**	**+**	**–**	**–**	**+**	**W**
**D-Raffinose**	**–**	**–**	**–**	**–**	**+**
**Gentiobiose**	**+**	**+**	**+**	**+**	**+**
**D-Tagatose**	**+**	**–**	**+**	**+**	**–**
**D-Arabitol**	**–**	**–**	**–**	**–**	**–**

TABLE S9 Differential phenotypic characteristics between isolates and closely related species. Kim37-2 (JCM 35346T), KimH (JCM 35345T), *Bifidobacterium aemilianum* (DSM 104956T), *Bombiscardovia coagulans* (DSM 22924T). +, Positive; –, Negative; w, weakly positive; NT, not tested. Ac, acetic acid; Lc, lactic acid. For Kim37-2, acid is produced from D-glucose, D-fructose, D-ribose, salicin, L-arabinose (weak), D-mannose (weak). No acid is produced from glycerol, erythritol, D-arabinose, esculin, D-galactose, amygdalin, L-sorbose, L-rhamnose, D-xylose, L-xylose , D-adonitol, dulcitol, inositol, D-sorbitol, methyl-D-xylopyranoside, methyl-D-mannopyranoside, methyl-D-glucopyranoside, D-maltose, D-lactose, D-melibiose, D-sucrose, inulin, D-melezitose, D-raffinose, starch, glycogen, xylitol, D-turanose, D-lyxose, D-fucose, L-fucose, D-arabitol, L-arabitol, gluconate, D-trehalose, D-mannitol , *N*-acetylglucosamine, arbutin, D-cellobiose, gentiobiose, D-tagatose, 2-keto gluconate, and 5-keto gluconate. For KimH, acid is produced from D-glucose, D-ribose, salicin, D-trehalose, L-arabinose (weak), gentiobiose (weak), D-fructose (weak). No acid is produced from glycerol, erythritol, D-arabinose, *N*-acetylglucosamine, esculin, D-galactose, D-cellobiose, amygdalin, L-sorbose, L-rhamnose, D-xylose, L-xylose , D-adonitol, dulcitol, inositol, D-mannitol, D-sorbitol, methyl-D-xylopyranoside, methyl-D-mannopyranoside, methyl-D-glucopyranoside, arbutin, D-maltose, D-mannose, D-lactose, D-melibiose, D-sucrose, inulin, D-melezitose, D-raffinose, starch, glycogen, xylitol, D-turanose, D-lyxose, D-tagatose, D-fucose, L-fucose, D-arabitol, L-arabitol, gluconate, 2-keto gluconate, and 5-keto gluconate.

	**Kim37-2**	**KimH**	***B. aemilianum***	***Bom. coagulans***
**GC content**	57.6	53.9	61.9	46.6
**Aerobic growth**	+	+	-	+
**Microaerophilic growth**	+	+	+	+
**Motility**	–	–	–	–
**Ratio of Ac : Lc**	1:1	1:1	NT	1:1
**Nitrate reduction**	–	–	NT	–
**Temperature range**	25-40	30-40	25-37	5-42
**F6PPK**	+	+	+	+
**Genome size**	2.3	1.9	2.0	1.7
Utilization of **:**				
**L-Arabinose**	W	W	W	+
**D-Xylose**	–	–	+	–
**D-Glucose**	+	+	+	+
**D-Fructose**	+	W	+	+
**D-Mannose**	W	–	+	–
**L-Rhamnose**	–	–	–	–
**D-Mannitol**	–	–	–	–
**D-Ribose**	+	+	+	+
**N-Acethyl Glucosamine**	–	–	+	–
**Amygdalin**	–	–	–	+
**Arbutin**	–	–	+	+
**Salicin**	+	+	+	+
**D-Cellobiose**	–	–	–	–
**D-Melibiose**	–	–	W	–
**D-Sucrose**	–	–	+	NT
**D-Trehalose**	–	+	–	+
**D-Raffinose**	–	–	+	+
**Gentiobiose**	–	W	–	+

TABLE S10 Cellular fatty acid content (%) of isolates from *Xylocopa* species.

Fatty acid	**KimC2**	**XA3**	**Kim32-2**	**Kim37-2**	**KimH**
16:0 FAME	42.08	31.11	15.43	42.64	27.86
18:0 FAME	-	2.74	2.84	6.21	7.21
16:1 CIS 9 FAME	-	1.56	-	0.85	-
18:1 CIS 9 FAME	27.37	32.18	71.88	17.57	33.24
18:1 CIS 11 DMA	-	1.98	-	-	-
19 CYC 9,10/:1 FAME	30.56	27.22	2.82	-	28.3
UN 18.199 18:Oa DMA	-	-	1.99	-	-
Summed feature 7	-	-	-	23.46	-
Summed feature 8	-	-	-	5.52	-
summed feature10	-	-	5.04	-	3.39
summed feature12	-	3.21	-	-	-

summed feature 7 : un 18.846/19:1 w6c
summed feature8 : 18:1 w7c
summed feature10 : 18:1c11/t9/t6 FAME
summed feature12 : 19:0 ISO FAME

TABLE S11 Summary of whole genome sequence obtained from the Pacific Bioscience (PacBio) and assembly.

Strain Name	Subread Bases	Subreads	Subread N50	Average Read Length	Average read coverage
KimC2	1,125,533,754	110,690	14,732	10,168	495
XA3	758,500,740	79,252	11,776	9,698	310
Kim32-2	872,777,133	92,471	11,316	9,438	497
KimH	849,623,299	91,005	11,534	9,472	415
Kim37-2	1,253,921,383	116,878	14,673	10,728	365
